# Use of Nanocarriers Containing Antitrypanosomal Drugs for the Treatment of Chagas Disease

**DOI:** 10.3390/ph16081163

**Published:** 2023-08-15

**Authors:** Diogo de Freitas Paiva, Ana Paula dos Santos Matos, Denise de Abreu Garófalo, Tatielle do Nascimento, Mariana Sato de Souza de Bustamante Monteiro, Ralph Santos-Oliveira, Eduardo Ricci-Junior

**Affiliations:** 1 Laboratory of Pharmaceutical Nanotechnology, Department of Drugs and Medications, Faculty of Pharmacy, Universidade Federal do Rio de Janeiro (UFRJ), Rio de Janeiro 21941-902, Brazil; di0g0fp2@gmail.com (D.d.F.P.); anapaulasmatos@gmail.com (A.P.d.S.M.); denise_gar@hotmail.com (D.d.A.G.); tatiellenascimento@hotmail.com (T.d.N.); mari-sato@hotmail.com (M.S.d.S.d.B.M.); 2 Nuclear Engineering Institute (IEN), University Campus of the Federal University of Rio de Janeiro, Rio de Janeiro 21941-906, Brazil; roliveira@ien.gov.br

**Keywords:** nanocarriers, Chagas disease, *Trypanosoma cruzi*, in vivo assays

## Abstract

Chagas disease, caused by the *Trypanosoma cruzi* parasitic protozoan, is a neglected tropical disease (NTD) of significant incidence in Latin America. Transmission to humans and other mammals is mainly via the vector insect from the Reduviidae family, popularly known as the kissing bug. There are other transmission means, such as through congenital transmission, blood transfusions, organ transplantations, and the consumption of contaminated food. For more than 50 years, the disease has been treated with benznidazole and nifurtimox, which are only effective during the acute phase of the disease. In addition to their low efficacy in the chronic phase, they cause many adverse effects and are somewhat selective. The use of nanocarriers has received significant attention due to their ability to encapsulate and release therapeutic agents in a controlled manner. Generally, their diameter ranges from 100 to 300 nanometers. The objective of this scoping review was to perform a search of the literature for the use of nanocarriers as an alternative for improving the treatment of Chagas disease and to suggest future research. Bibliographic searches were carried out in the Web of Science and PubMed scientific databases from January 2012 to May 2023, using the “Chagas disease and *Trypanosoma cruzi* and nanoparticles” keywords, seeking to gather the largest number of articles, which were evaluated using the inclusion and exclusion criteria. After analyzing the papers, the results showed that nanocarriers offer physiological stability and safety for the transport and controlled release of drugs. They can increase solubility and selectivity against the parasite. The in vitro assays showed that the trypanocidal activity of the drug was not impaired after encapsulation. In the in vivo assays, parasitemia reduction and high survival and cure rates in animals were obtained during both phases of the disease using lower doses when compared to the standard treatment. The scoping review showed that nanocarriers are a promising alternative for the treatment of Chagas disease.

## 1. Introduction

Chagas disease, or American trypanosomiasis, was discovered by the Brazilian Carlos Chagas in 1909 [1]. It is an infection caused by the *Trypanosoma cruzi* parasitic protozoan. The World Health Organization (WHO) estimates that there are from 6 to 8 million people infected with the parasite in the world, with a higher incidence in endemic areas of South American countries [2,3]. Approximately 10,000 people die every year because of the disease [4,5]. It is considered a neglected tropical disease (NTD) due to the pharmaceutical companies lacking interest in investing in the search for new treatments and increasing professional specialization for faster and more efficient diagnoses. Despite that, the disease is no longer exclusive to developing countries [1]. Due to the increase in immigration to other countries, many cases are diagnosed in European countries, Canada, Australia, Japan, and the United States [2,4,6].

The main transmission means is vectorial by blood-sucking insects of the Reduviidae family, popularly known as the kissing bug [7]. When the insect bites, it defecates in the region close to the bite, generating itching. This makes the person scratch the area, taking feces and urine containing the infective form of *T. cruzi* inside the wound [4,6]. Other transmission means are already known, through blood transfusions in banks that do not carry out screening, organ transplants, congenital transmission, and oral transmission by the consumption of food or beverages contaminated with the infected vector [1,6].

*Trypanosoma cruzi* presents in three different forms in its development cycle and needs two different types of hosts: invertebrate ones (hematophagous insects) and vertebrate ones (mammals) [6,8]. The epimastigote form is found in the vector, replicative but non-infectious. The circulating trypomastigote form is found in the hosts; it is infectious and not replicative. The intracellular amastigote form is only replicative and is present in vertebrate hosts [9]. When the vector bites a contaminated host, it ingests blood in the metacyclic trypomastigote form, which in the invertebrate organism will differentiate into epimastigotes that multiply by binary fission [6,9]. Subsequently, these epimastigote forms are differentiated into trypomastigotes that will be released in triatomine excretions. Upon entering the wound of the human host, the trypomastigote form penetrates the cells and transforms into amastigotes, which will multiply and differentiate into trypomastigotes through several binary division cycles until causing the cell to break, which releases amastigotes and trypomastigotes, infecting neighboring cells and spreading [9]. The infective forms of the parasite can penetrate any type of nucleated cell, including defense cells such as macrophages [1,4]. They present tropism via myocytes, which explains their presence in cardiac muscle cells causing heart dysfunction [1].

Chagas disease is divided into two clinical phases: acute and chronic. The acute phase can manifest early in time or progress to being undetermined, silent, and asymptomatic. In the early acute stage, the disease can disappear spontaneously without causing complications in most infected individuals due to adaptive immunity control or present with nonspecific symptoms [1,2]. It can be characterized by the onset of inflammation in the parasite entry region and present with more ease of parasitemia detection [2,6]. In the undetermined silent and asymptomatic phase, the parasites hide in different tissues, hindering diagnosis [2]. When progressing to the chronic phase of the disease, the patient has a 30% to 40% chance, after years or decades, of developing megaesophagus, megacolon, and chronic chagasic cardiomyopathy, which is the most severe form of the disease and, without treatment that can revert the clinical picture, possibly presenting with thromboembolism, cardiac arrhythmias, and cardiac arrest [1,2,6,8]. 

**Figure 1:**
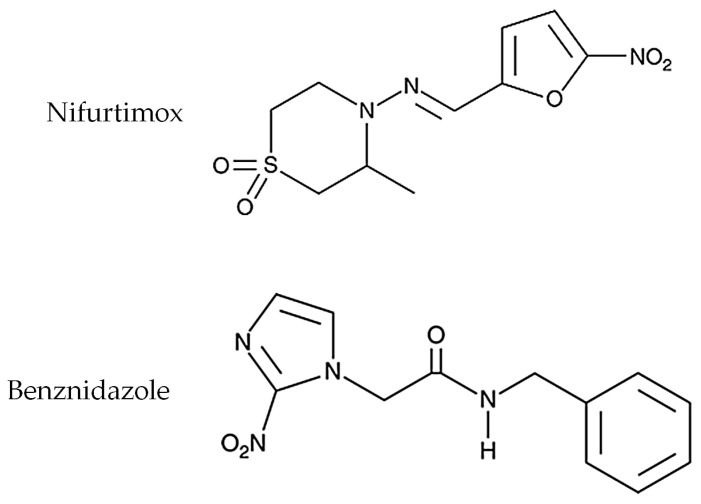
Chemical structures of nifurtimox and benznidazole.

Since the 1960s, the only treatments known for Chagas disease have been two nitro-heterocyclic compounds: nifurtimox and benznidazole (Figure 1) [3,9]. Despite a therapeutic efficacy of up to 80% presented during treatment in the acute phase, the drugs have low efficacy in the chronic phase [1,3]. In addition, they have high toxicity, low solubility in aqueous media, difficulty crossing biological barriers, low selectivity, and serious adverse effects, leading many patients to abandon drug treatment [3,8,9]. Such high toxicity can be related to the high doses required for the drugs to achieve the effective concentration to cause intracellular death [1]. 

The treatment with nifurtimox causes stronger adverse effects than using benznidazole, even when administered in low doses. The most frequent adverse effects are gastrointestinal symptoms, anorexia, sleepiness, and paresthesia [9].

Benznidazole is the first-choice drug for treating the infection, despite the low cure rate in the chronic phase of the disease [3]. Its main adverse effects are peripheral neuropathy, cutaneous manifestations, paresthesia, anorexia, gastrointestinal symptoms, and more severe ones such as decrease in bone marrow and agranulocytosis [9,10]. Other factors can influence the outcome of the treatment with benznidazole, such as the different parasite strains, emergence of drug-resistant strains, the patient’s age, the progression of the disease, the long period required for the treatment, and the lack of appropriate infant formulations [6,9,10].

The WHO has determined that the ideal drug for treatment should present a parasitological cure and show dose efficacy during the acute and chronic phases, not present adverse effects, or cause resistance, or induce teratogenic effects. The problem is that no currently known drug manages to cover all these needs [9].

Due to the limitations presented by the only treatments currently known to combat the Chagas disease infection, it has become necessary to search for and learn about new therapeutic options that can improve the desired parameters.

In recent years, nanotechnology has been a promising option for studies aimed at finding ways to improve the treatments of various diseases. It is a type of technology that uses nanoscience practically with multiple functions performed by structures at a nanometric scale, from 1 to 100 nm [11,12]. Nanoparticles can improve bioavailability, promote sustained release, and decrease toxicity. Nanoparticles can stimulate immune system cells, such as macrophages, promoting the elimination of intracellular parasites [13]. Nanoparticles can bind to receptors located on the surface of cells, resulting in cellular uptake [14]. 

Drugs and nanocarriers can cross the cell membrane by endocytosis or direct permeation. In direct permeation, molecules and particles cross the cell membrane by a mechanism controlled by diffusion or pore formation. However, nanocarriers with polar surfaces and hydrophilic drugs do not cross the plasma membrane easily with a lipophilic nature. To cross the cell membrane, hydrophilic drugs and nanocarriers of polar nature need active transport that involves energy expenditure, such as endocytosis, in which the cell captures extracellular materials through invaginations of the membrane to form endosomes. There are two forms of endocytosis: phagocytosis and macropinocytosis. In phagocytosis, nanomaterials are captured by activated phagocytes through the process of opsonization, in which opsonins, such as immunoglobulins and complement system proteins, coat the target particle. In macropinocytosis, the cell captures liquids and solids (nanoparticles and nanocarriers) with the formation of vesicles called pinosomes. Endocytosis can occur by clathrin-mediated endocytosis, caveolin-mediated endocytosis, and clathrin–caveolin independent endocytosis. Thus, the cellular internalization of nanocarriers depends not only on physicochemical properties, such as the size, shape, surface charge and polarity, but also on the presence of ligands on the surface [15].

**Figure 2:**
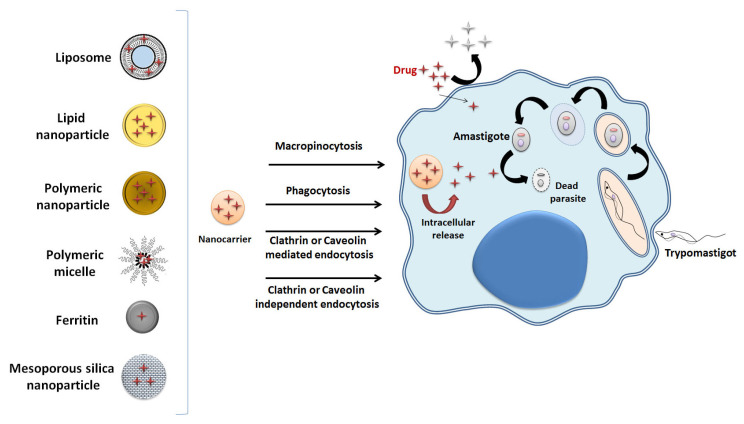
Nanocarriers used for the treatment of Chagas disease and cell uptake routes.

Nanoparticles can be classified into polymeric, lipid, metallic, and mesoporous silica (Figure 2) [13]. Lipid nanoparticles are divided into two types: solid lipid nanoparticles (SLNs), and nanostructured lipid carriers (NLCs). Due to the presence of lipids, SLNs are biocompatible and biodegradable and present high bioavailability. They are composed of the same mixture of liquid and solid lipids. Their rigidity confers better protection to the incorporated drug. They are fairly stable, produced on a large scale, and can encapsulate drugs to provide controlled release [6,13,14]. NLCs are like SLNs but can incorporate various types of drugs and liquid lipids in their internal parts [14]. 

Polymeric nanoparticles are solid colloidal particles generally composed of biodegradable and biocompatible polymers approved by the US regulating agency, the Food and Drug Administration (FDA). They can be dissolved, encapsulated, or absorbed in the constituent polymeric matrix in systems for the release of therapeutic agents and be used as adjuvants in vaccines. They can be produced using countless types of natural or synthetic polymers, such as poly (lactic co-glycolic acid) (PLGA); polylactic acid (PLA); polyglycolide acid (PGA); polycaprolactone (PCL); chitosan; and polyethylene glycol (PEG) [6,13].

Mesoporous silica nanoparticles are potent drug releasers formed by a complex organized network of pores with homogeneous sizes and can assist in the functionalization of drugs. The metallic nanoparticles are formed by clusters of metal atoms [6,13].

In addition to nanoparticles, other nanocarriers are also widely used for the controlled release of drugs, such as liposomes and polymeric micelles. Liposomes are vesicular systems composed of one or more lipid bilayers of a spherical format with sizes ranging from 50 to 500 nm. They are employed extensively due to their ability to encapsulate drugs, thanks to their hydrophobic and hydrophilic characteristics, high bioavailability, and biocompatibility [16]. 

Polymeric micelles are amphiphilic polymer structures that have a hydrophobic part and a hydrophilic component. They can have different formats and are prepared in various ways. Among their advantages as drug vehicles, they have a nanoscale size, selective targeting, storage stability, and reduction in adverse effects [17].

Also, cage-like proteins such as Ferritin can be used as drug carriers [18]. Ferritins can be particularly important in delivering drugs to cure leishmaniasis since ferritins are phagocytized by the macrophages where the parasites live and multiply [19].

The objective of this scoping review is to identify and analyze knowledge gaps and examine how research is conducted in relation to the use of nanocarriers containing trypanocidal drugs to treat Chagas disease, highlighting articles with in vivo studies. This scoping review differs from the others because it will search scientific databases for articles focusing on in vivo studies containing robust in vitro studies published in the last ten years, using nanocarriers as an alternative for the treatment of infection caused by the *Trypanosoma cruzi* parasite.

## 2. Results

### 2.1. Bibliographic Research of Articles in the Databases

The number of articles found during the research in the databases chosen to prepare this paper is presented in Table 1.

Added to the results of the searches carried out in the PubMed and Web of Science databases covering the period from January 2012 to May 2023, 123 articles were found with the “Chagas disease and nanoparticles” keyword, 120 for “*Trypanosoma cruzi* and nanoparticles”, and 87 using “Chagas disease and *Trypanosoma cruzi* and nanoparticles”. All 87 articles found in the research using the “Chagas Disease and *Trypanosoma cruzi* and nanoparticles” keywords were selected to proceed to the following stage.

**Figure 3:**
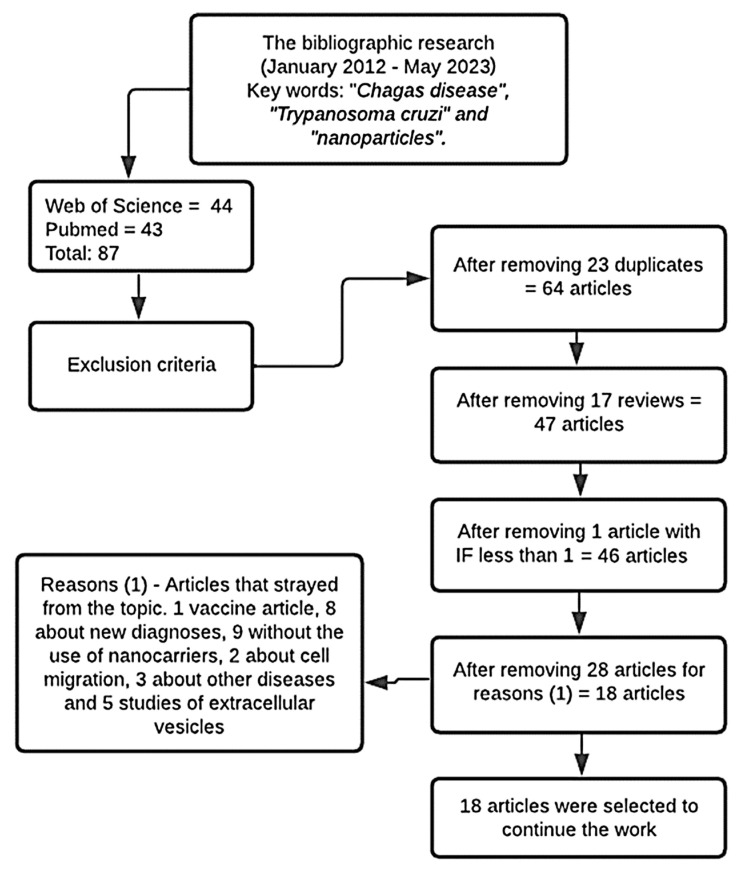
Flowchart corresponding to selection of the articles for the bibliographic review, as PAGE et al. [20].

All 87 articles were analyzed according to the exclusion criteria, removing 23 duplicates and 17 review articles. No congress abstracts, patents, or book chapters were found. Only one article was excluded for having been published in a journal with an impact factor below 1.0. The remaining 46 papers had their content assessed by reading, excluding those that did not deal with the topic of interest. A total of 28 articles were removed; among them: one article on vaccines, eight articles on new diagnostic techniques for detecting the disease, nine articles that did not use nanocarriers, two studies on cell migration, three studies that addressed other diseases such as leishmaniasis and toxoplasmosis, and five studies on extracellular vesicles (Evs). In the end, 18 articles were selected for review. The process to select the articles is shown in the flowchart in Figure 3.

The assessment of the remaining 18 articles is presented in Table 2.

As shown in Table 2, two articles [1,10] obtained the maximum score, as they presented all the topics selected for the assessment. The article that obtained the lowest score was [3], as it only conducted in vitro assays, obtaining a null score in all the topics related to in vivo assays, in addition to not presenting other issues such as physicochemical characterization and the nanometric size of the nanocarrier. The articles [2,3,5,22,23,24] received a zero for the topics of ethics statement, study design, animals, sample size, and in vivo studies, as they did not present in vivo studies, only in vitro ones.

The articles that presented in vivo tests were [1,7,8,10,25,27,28,29,30,31]. 

All the articles were scored for presenting full abstracts, contextualization, and objectives in their introduction and interpretation of the results with a conclusion [1,2,3,4,5,7,8,10,22,23,24,25,26,27,28,29,30,31]. 

The article of Abriata [29] had the topic of the title scored as zero, as it did not specify the content of in vitro assays, even if present in the paper. Nhavene [3] was the only article that did not report the nanometric scale size of the nanoparticle. In the article of Branquinho [31], the complete physicochemical characterization had been previously performed by Branquinho [32]. Some articles did not present the characterization of the morphology [4,7,8,22,26,27,28,30] and the polydispersity index [3,22,23,25] of the nanoparticles; therefore, they obtained a zero in physicochemical characterization.

Only the article [2] did not present an efficient trypanocidal effect in the tests with the nanoparticles and, along with articles [8,23], obtained zero points on the topic of limitations. The only article that did not present any statistics was [3].

### 2.2. Nanocarriers

The composition and characterization of the structures of the nanocarriers used are shown in Table 3.

**Figure 4:**
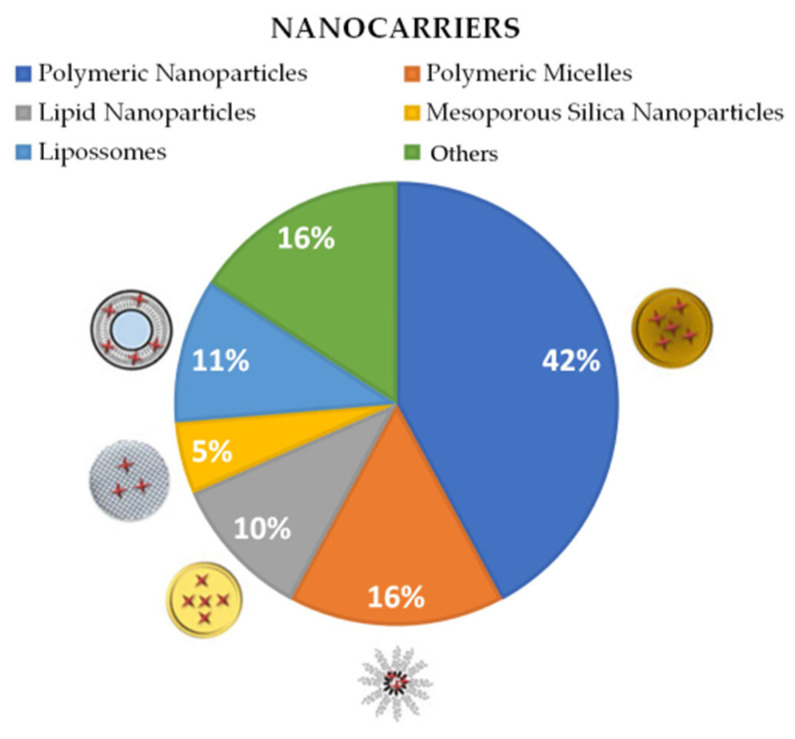
Types of nanocarriers used for delivery of anti-*T. cruzi* drugs. Others: metallic nanoparticles, composite nanoparticles, and polymerosomes.

Most of the research used polymeric nanoparticles as drug nanocarriers [4,7,8,24,25,29,30,31], followed by polymeric micelles [26,27,28], and by solid lipid nanoparticles [2,10]. In addition to solid lipid nanoparticles (SLN), the article by Vinuesa [2] evaluated nanostructured lipid carriers (NLCs) and liposomes in the tests. Of the selected articles, only one employed silver nanoparticles [5], and another resorted to mesoporous silica nanoparticles [3]. One of the articles applied the organic nanoparticle system called MOF (Metal–Organic Framework), composed of hybrid polymers [23]. The article by Tessarolo [22] presented an inorganic CaCO_3_ particle containing encapsulated BZN. Li [1] worked with vesicular nanocarriers known as polymerosomes. The types of nanostructures used in the papers are represented in Figure 4. 

### 2.3. Components

To produce polymeric nanoparticles, among the components used in the studies there are PLGA [25], different types of polyethylene glycol (PEG) [7,8,30,31], PCL [29,30,31], and poloxamer [7,29,30,31]. In the papers that produced lipid nanoparticles or lipid carriers, among the components used are poloxamer 188, polysorbate 80, sodium taurodeoxycholate, Precirol^®^, and Miglyol^®^ [2,10]. For the silver nanoparticles, silver nitrate and *Iresine herbstii* leaves were used to extract the natural active substance [5]. Chitosan was used in the paper that employed the chitosan polymeric nanoparticle [4] and in the one that produced the mesoporous silica nanoparticle [3]. To produce the hybrid polymer nanoparticles, metal–organic frameworks (MOFs) were used [23]. Poloxamer 188 (Lutrol F-68) was also used as a stabilizer in the synthesis of polymeric micelles [26,27,28]. PEG thioacetate and mesylate were used in the production of the polymersome [1].

Metal–organic frameworks (MOFs) are hybrid polymers belonging to a new class of nanocarriers of drugs. They can be produced at a nanoscale and their use presents benefits such as a greater surface area at the nanoscale, porosity, and crystallization, which improves the interaction potential with other molecules [23].

The use of chitosan in the articles [3,4] is explained by its biodegradability, biocompatibility, structural stability, low toxicity, and cationic nature, which result in good interaction with cell membranes and increased endocytosis. It was quite often used in drug carriers for biomedical systems in the articles [4,33]. 

PCL was used for the formation of polymeric nanoparticles and is a synthetic polymer used as a carrier for controlled drug release [29,30,31]. It is widely applied because it is designed to have good biocompatibility and biodegradability [29].

### 2.4. Anti-T. cruzi Drugs

The drug used by most of the studies was benznidazole [1,2,3,22,25,26,27,28], which is the first-choice medication for the treatment of Chagas disease. One other drug frequently studied was a natural product, lychnopholide (LYC), a sesquiterpene lactone extracted from *Lychnophora trichocarpha* with anti-*T. cruzi* activity previously reported by De Oliveira [34]. It was employed in the papers [7,8,30,31]. In addition to LIC, other natural active molecules were also used, such as corn cob xylan, a bioactive polysaccharide extracted from the *Iresine herbstii* leaves [5]; curcumin, a polyphenolic flavonoid with anti-inflammatory, antioxidant, and immunomodulatory properties [25], which was combined with benznidazole in the study by Hernández [25]; and ursolic acid, a natural pentacyclic triterpene that has already shown efficacy in the treatment against *Trypanosoma cruzi* infection in vivo [29].

**Figure 5:**
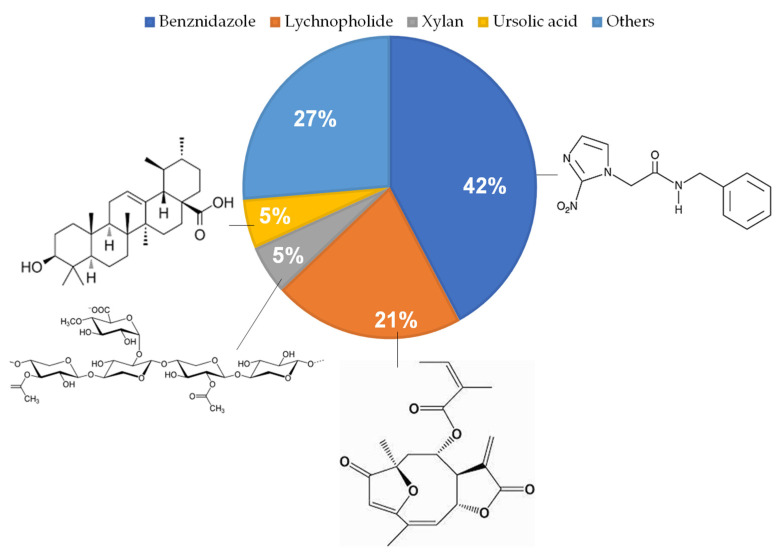
Anti-*T. cruzi* drugs encapsulated in nanocarriers. Others: Curcumin, ergosterol peroxide, sodium diethyldithiocarbamate, 5-hydroxy-3-methyl-5-phenyl-pyrazoline-1-(S-benzyl dithiocarbonate) (H2bdtc), and mercaptosuccinic acid with nitric oxide (NO).

In addition to these compounds, ergosterol peroxide was also used, which, according to data previously obtained, has trypanocidal activity in vitro [23] and sodium diethyldithiocarbamate, which has already shown activity against *T. cruzi* in studies, by chelating metals and stimulating reactive oxygen species (ROS), causing damage to the parasites [24]. In addition, 5-hydroxy-3-methyl-5-phenyl-pyrazoline-1-(S-benzyl dithiocarbonate) (H2bdtc) was used, a cyclic compound derived from S-dithiocarbonate and 1,3-diketones, with significant trypanocidal activity [10] and mercaptosuccinic acid with nitrosation to release nitric oxide (NO), which may be able to kill the parasite and control disease progression [4]. Figure 5 presents a scheme of anti-*T. cruzi* drugs encapsulated in nanocarriers.

### 2.5. Preparation Method

Most of the studies used the interfacial polymer deposition method after solvent displacement. It is a conventional method for preparing nanoparticles and is also called solvent diffusion or nanoprecipitation [7,8,24,26,27,28,29,30,31]. The articles [8,26,31] reported that the method had been previously described by Scalise et al. [28], Branquinho et al. [35], and Branquinho et al. [32]. The emulsification method was employed in the papers [22,25]. Vinuesa et al. [2] used the hot homogenization technique to produce NLCs, emulsification for SLNs, and the fluid compression technique, called DELOS-SUSP, for liposomes.

**Figure 6:**
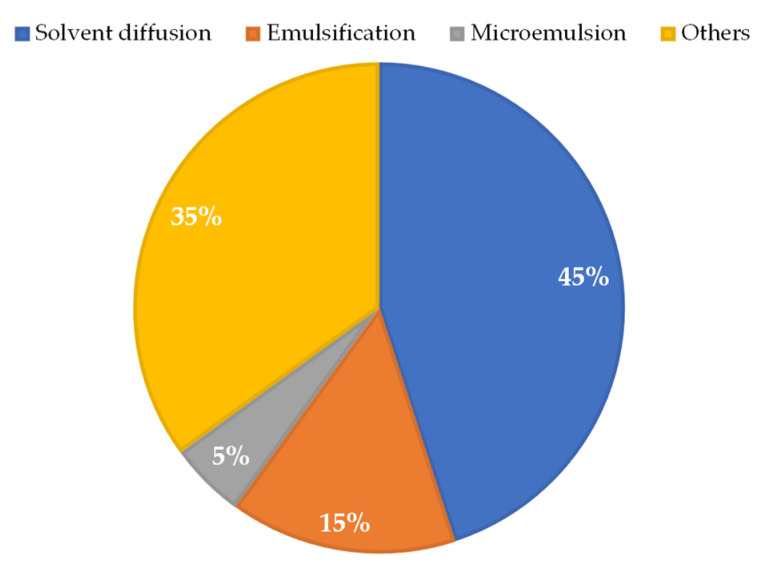
Methods used by the papers in the syntheses of nanocarriers. Others: Hot homogenization technique, emulsion techniques (O/W), heating and cooling in the formation of crystals, sensitized photo-oxygenation in methanol with eosin, mechanochemistry, ionotropic gelation, anionic polymerization with methanol precipitation and thin film rehydration, green synthesis, condensation hydrolysis and the fluid compression technique called DELOS-SUSP.

For the synthesis of MOF nanoparticles, the article by Morales-Baez [23] used heating and cooling in crystal formation. For the synthesis of ergosterol peroxide, sensitized photooxygenation in methanol with eosin was used. Finally, mechanochemistry was employed for coupling. In other studies, microemulsion [10], ionotropic gelation with agitation and suspension for chitosan nanoparticles [4], anionic polymerization with precipitation in methanol and thin film rehydration [1], and green synthesis process with continuous agitation for the production of silver nanoparticles [5] were used. In the synthesis of mesoporous silica nanoparticles, the study [3] followed the procedure previously described by Fan et al. [36] in which a model of positively charged N-cetyltrimethylammonium bromide (CTAB) and a NaOH catalyst were used through hydrolysis and condensation. The methods used in the papers are summarized in Figure 6.

### 2.6. Physicochemical Characterization

All the papers reported the nanometric size achieved [1,2,4,5,7,8,10,22,23,24,25,26,27,28,29,30,31], except [3]. The techniques used for size characterization were atomic force microscopy (AFM) [5,22,24,31], dynamic light scattering (DLS) [1,2,4,5,7,8,24,25,27,29,30,31], scanning electron microscopy (SEM) [5,23], and photon correlation spectroscopy (PCS) [10,26,28].

For the morphology of the nanocarriers, the papers used transmission electron microscopy (TEM) [1,3,23], SEM [2,29], and AFM [10,24,25,31] techniques. The other articles did not present the morphology of the nanocarriers [4,7,8,22,26,27,28,30].

Zeta potential was obtained by DLS [1,2,3,4,5,27,31], AFM [25], electrophoretic mobility [10,24,26,28], electrophoretic light scattering (ELS) [29], and laser Doppler anemometry associated with microelectrophoresis [30]. The articles [7,8,23] did not present the zeta potential of the nanocarriers. Tessarolo et al. [22] did present the zeta potential value, although without specifying the technique used. 

For the polydispersity index (PDI), most articles used the DLS technique [1,2,4,5,7,8,24,27,29,30,31]. The PCS technique was used by Carneiro et al. [10] to determine the PDI. Rial et al. [26] and Scalise et al. [28] presented the PDI values without specifying the technique used. The articles [3,22,23,25] did not present the PDI in their studies. Some articles also showed results of the encapsulation efficiency (EE) characterization [1,2,4,10,24,29] and loading efficiency [1,24] of the nanostructures using UV-Vis spectrophotometry and high-efficiency liquid chromatography (HPLC) techniques.

For the quantification of the drug and analysis of coupling with the nanocarrier, infrared (IR) spectroscopy [22,23] and X-ray diffraction (XRD) [23] were used. Vinuesa et al. [2] evaluated the cumulative release for SLNs and NLCs and Abriata et al. [29] obtained the thermal behavior by differential scanning calorimetry (DSC). For the surface characterization of nanocarriers and copolymers, solid-state nuclear magnetic resonance (SS NMR) was used in the studies [1,3]. Nhavene et al. [3] also characterized the elemental composition by energy-filtered transmission electron microscopy (EFTEM), the quantification of organic molecules by elemental analysis (CHN), and the chemical composition and binding state of the elements on the surface of the nanoparticles by X-ray photoelectron spectroscopy (XPS). Other techniques used in the characterization studies were Fourier transform infrared spectroscopy (FTIR) [5], infrared absorption spectroscopy (FTIR-ATR) [24], high-performance liquid chromatography with ultraviolet detection (HPLC-UV) [31], and content (DL) [10,24]. Brito et al. [5] also used Raman spectroscopy, energy dispersion X-ray spectroscopy (EDS), and inductively coupled plasma optical emission spectroscopy (ICP-OES) for the characterization.

### 2.7. In Vitro Assays

Table 4 presents the in vitro assays and their respective results found by the studies analyzed in this review. The papers selected for the bibliographic research that are not included in the table did not conduct in vitro assays [8,25,26,30,31].

The studies used different cell types to conduct the in vitro assays. The cell lines used were LLCMK2 (Rhesus monkey renal epithelial cell) [4,10,22,29], NIH-3T3 (isolated mouse fibroblast cell line) [5,23,24], J774A.1 (monocyte, mouse macrophage) [23], Vero (African green monkey renal epithelial cells) [23,24,27,28], L-929 (murine fibroblasts) [2], Hep G2 (human hepatocellular cell line) [2], H9C2 from mouse myoblasts [1], and RAW (derived from macrophages) [24]. Peritoneal macrophage cell lines [4], cardiomyocytes from healthy mice [7], and spleen cells isolated from C57BL/6 mice [10] were also used. 

The test most employed in the articles was MTT, which measures the cytotoxicity of the nanoparticles conferred to the cells, through extracellular reduction of the tetrazolium salts [4,5,22,23,24,28]. The study [29] assessed cytotoxicity using the resazurin method. Carneiro et al. [10] resorted to flow cytometry to assess cytotoxicity. Branquinho et al. [7] evaluated the toxicity potential of the drug through calcium homeostasis in healthy mouse cells. Vinuesa et al. [2] used the water-soluble tetrazolium (WST) assay to assess cytotoxicity. The studies [1,3] did not employ any cell cytotoxicity assay. The paper by Rial [27] performed tests to quantify ROS and the production of specific *T. cruzi* antibodies in cardiac tissue inflammation.

The papers described tests to evaluate the trypanocidal effect of drugs in free form and of the nanocarriers against the different forms of the evolutionary cycle of *Trypanosoma cruzi*. For the epimastigote, trypomastigote, and amastigote forms, tests of trypanocidal activity at different concentrations, growth inhibition assays, and effect on viability and efficacy were applied [1,2,3,4,5,10,22,23,24,28]. Many of the papers selected did not perform tests for the amastigote form [3,4,5,7,10,23,24,27,29]. The articles [3,5] only carried out tests for the epimastigote form, a non-infective form, present in the invertebrate vector. Scalise et al. [28] only employed the growth inhibition assay in the intracellular amastigote form. For more robust results, all studies should present tests against the three forms of *Trypanosoma cruzi*; mainly against circulating trypomastigotes and intracellular amastigotes, because they are the forms present in vertebrates. The lack of these tests is considered a limitation of the studies.

Few studies presented results for CC_50_, which assesses the drug and nanoparticle concentrations required to kill 50% of the uninfected cells [4,22,23]. The articles [2,22] obtained results of the drug and nanoparticle concentrations required to kill 50% of the parasites present in the infected cells (IC_50_) in all three *T. cruzi* forms. Morales-Baez et al. [23] and Contreras Lancheros et al. [4] did not perform tests to obtain IC_50_ in amastigotes, just as Li et al. [1] did not obtain it for the epimastigote form. The articles [10,24] presented the IC_50_ result only for the trypomastigotes forms. The papers [3,5,7,27,28,29] did not present CC_50_ or IC_50_ data.

Some articles calculated the selectivity index (SI), which evaluates the activity of the compound against infected cells, without causing cellular cytotoxicity in the host [2,4,22]. Understanding the degree of cytotoxicity of free drugs and drugs encapsulated in nanocarriers is important to assess the impact on parasite elimination without harming host cells. Thus, it is essential to calculate the selectivity index (SI), which can be obtained through CC_50_ and IC_50_ studies.

### 2.8. In Vivo Assays

Table 5 lists the in vivo assays conducted with animals infected and not infected with *Trypanosoma cruzi* by the papers selected in this bibliographic review. The studies that did not conduct in vivo assays are not included in Table 5 [2,3,4,22,23,24,27].

To carry out the in vivo tests, the researchers used the following animal models: C57BL/6 mice [7,25,26,29], BALB/c mice [1], C3H/HeN mice [27,28], and Swiss mice [8,10,30,31] aged around 1-month-old and of both sexes. The study [29] was the only one that did not report the animals’ age. The *T. cruzi* infection in animals was carried out intraperitoneally in all studies [1,8,10,25,26,27,28,29,30,31]. Branquinho et al. [7] did not provide information on the route used to infect the animals. All animals were infected by the trypomastigote form of *Trypanosoma cruzi*, which is the circulating and infective form of the parasite.

The studies used the infected and untreated animals as the control group [1,7,8,10,26,27,28,29,30,31]. Hernández et al. [25] used a group of untreated healthy animals and another group of untreated infected animals as control groups. 

The studies [1,8,27,28,29] conducted the animals’ treatment during the acute phase of the disease. The studies [25,26] conducted the treatment in the chronic phase. In turn, the papers [7,30,31] applied the treatment during both phases of the infection. Carneiro [10] did not report the phase in which the treatment was conducted. For the administration of drugs and nanoparticles, many studies used the oral route by gavage [1,10,25,26,27,28,30,31]. The papers [1,30] were also administered via the intravenous route; in turn, those by Branquinho et al. [7,8] only resorted to the intravenous route. 

After the treatment, the main assays applied were to assess the parasitemia level [1,8,10,25,26,27,28,29,30,31] and the survival rate [27,28,30,31]. In addition, assays for the detection of cardiac enzymes [10,25] and histological analysis of the heart [27,28,30,31] were used.

## 3. Discussion

### 3.1. In Vitro Data Discussion

The discussion was carried out after analyzing the in vitro results of the articles selected in the Table 4.

#### 3.1.1. Polymeric Nanoparticles

Polymeric nanoparticles were widely used by the articles in this review, although only Contreras Lancheros [4] and De Freitas Oliveira [24] showed better results as compared to the free drug. 

Contreras Lancheros et al. [4] synthesized chitosan polymeric nanoparticles containing NO. During the infection by the parasite, macrophages release nitric oxide. It is believed that these molecules are responsible for inhibiting the replication of the parasite. The problem is that NO is short-lived and can be toxic to some tissues [4]. The study coupled mercaptosuccinic acid (MSA), an NO precursor molecule, into polymeric nanoparticles with subsequent nitrosation by adding an equimolar amount of sodium nitrite (NaNO_2_) to form S-nitroso-MSA-CS NPs (NO-releasing nanoparticles). The paper evaluated cellular cytotoxicity in peritoneal macrophages and the effects of the nanoparticles against the *T. cruzi* forms, finding the following results: in the cellular viability evaluation by the MTT method, the CC_50_ value found was 400 ± 5.7 μg/mL after 72 h of exposure to the MSA polymeric nanoparticle (S-nitroso-MSA-CS NPs). For the different forms of *T. cruzi*, tests of effect against parasite proliferation were performed by direct count in a hemocytometer under a light microscope, finding IC_50_ values of 75.0 ± 6.5 μg/mL for epimastigotes and 25.0 ± 5.0 μg/mL for circulating trypomastigotes. The nanoparticles were also effective in reducing the intracellular amastigote forms. With these results, it was possible to carry out the calculation to obtain the selectivity index (SI), which was 5.3 for epimastigotes and 16 for trypomastigotes. The result was positive because it indicates greater selectivity of the nanoparticles by the parasite and sustained NO release when compared to the free donor [4]. 

De Freitas Oliveira et al. [24] synthesized polymeric nanoparticles of sodium diethyldithiocarbamate (DETC), a compound from the class of carbamates previously studied by the authors with promising results in combating Chagas disease. Its mechanism of action consists of stimulating ROS and chelating metals, which are harmful to the parasite. However, they can be harmful to the host. To avoid toxic effects and maintain an action against the parasite, the study encapsulated diethyldithiocarbamate in PLA [24]. The authors carried out tests to evaluate cytotoxicity by MTT in three different cell lines and the antiparasitic activity of the DETC nanoparticles against the circulating trypomastigote form. Free BZN was used as a positive control. When tested by different concentrations of the nanoparticles containing diethyldithiocarbamate in the cytotoxicity assays, all three cell lines: RAW, derived from macrophages; 3T3, derived from fibroblasts; and Vero, derived from renal epithelial cells, remained constant in viability. There was a significant reduction in viability only in the Vero cells, by 60%, at the highest concentration tested: 132 μM. When evaluating the antiparasitic activity, the study obtained IC_50_ results of the diethyldithiocarbamate nanoparticles for three different *T. cruzi* strains and compared them to the results found with free BZN [24]. For the Dm28c strain, the IC_50_ of the nanoparticles was 15.47 ± 2.71 μM, while the one for free BZN was 70.58 ± 6.87 μM. In the evaluation of the Y strain, IC_50_ was 45.15 ± 5.44 μM and 85.24 ± 5.22 μM for the diethyldithiocarbamate nanoparticles and for free BZN, respectively. For the Bolivia strain, the IC_50_ of the nanoparticles was 47.89 ± 3.98 μM and, for free BZN, it was 79.78 ± 6.18 μM. As can be seen, for all three strains of *Trypanosoma cruzi*, the IC_50_ was lower when using the diethyldithiocarbamate nanoparticles when compared to free BZN. The study did not present the selectivity index (SI) but concluded that the DETC nanoparticles demonstrated low toxicity when compared to the drug in free form due to its capacity for controlled release, reducing cell damage. The nanoparticles showed an IC_50_ similar to the one already found by the authors with the free drug in previous studies, which shows that there was no reduction in activity against the parasite in the encapsulated form with the controlled release [24].

#### 3.1.2. Lipid Nanoparticle

Several studies compared trypanocidal effects and cytotoxicity using another type of nanostructure: lipid nanoparticles. Vinuesa et al. [2] resorted to assays with different concentrations of SLNs, NLCs, and free BZN against all three forms of the *T. cruzi* cycle. For the circulating trypomastigote and intracellular amastigote forms, the IC_50_ values found were 17.6 ± 3.3 μM for NLCs, and 0.8 ± 0.4 μM for free BZN. For the epimastigote forms, the IC_50_ results were between 48.8 ± 14.3 μM and 123.9 ± 19.7 μM with SLNs, 41.3 ± 9.9 μM and 256.0 ± 19.9 μM for NLCs, and 17.7 ± 2.1 μM for free BZN. As can be seen, the values found for free BZN were better than with the use of lipid nanoparticles. This is also reflected by the numbers presented for the selectivity index, which were 21 and 69 for epimastigotes and trypomastigotes, respectively, with the use of NLCs, and 54 for epimastigotes and 904 for trypomastigotes with free BZN. In the cytotoxicity evaluation by WST performed in murine fibroblasts (L 929) and human liver cells (Hep G2), the article found mixed results for SLNs, requiring a reassessment of the accuracy of the test, which caused these nanoparticles to be removed from the study [2]. 

Carneiro et al. [10] compared in vitro the trypanocidal effects of the free compound H2bdtc (5-hydroxy-3-methyl-5-phenyl-pyrazoline-1-(S-benzyl dithiocarbonate)) with its conjugated form in a solid lipid nanoparticle (H2bdtc-SLNs) and with free BZN against the circulating trypomastigote form. With free H2bdtc, the IC_50_ found was 0.50 ± 0.12 μM, for the solid lipid nanoparticle loaded with H2bdtc it was 1.83 ± 0.18 μM, and with free BZN, the IC_50_ was 0.50 ± 0.39 μM. Cytotoxicity was evaluated with cells isolated from C57BL/6 mice. No significant cytotoxicity was found with the free and encapsulated form of H2bdtc. Despite the trypanocidal effect of free and H2bdtc-loaded in SLNs being like that of free BZN, the drug has a positive result, as it was not toxic to the cells [10].

#### 3.1.3. Other Nanoparticles

Tessarolo et al. [22] synthesized inorganic calcium carbonate nanoparticles loaded with 1 mg/mL of benznidazole (BZN encapsulated in CaCO_3_ nanoparticles) using the emulsification method. The use of calcium carbonate to produce the nanoparticle was due to its use in previous studies in the selective and targeted release of chemotherapy drugs in cancer treatments. The study used in vitro cytotoxicity and effect tests against all three forms of *T. cruzi* with the use of free BZN and calcium carbonate nanoparticles loaded with BZN at different times and concentrations, for comparison purposes. In the MTT cytotoxicity evaluation, the study used LLCMK2 mammalian cells. CC_50_ was 55.35 ± 9.03 µg/mL for BZN-loaded calcium carbonate nanoparticles and 160.4 ± 75.09 µg/mL for free BZN. The results show an increase in the cytotoxicity of BZN encapsulated in nanoparticles concerning the free drug, but that was not reflected in the SI, as the SI of encapsulated BZN was 30.5 and the one for free BZN was 2.38. The higher the SI value, the more selective the compound is for the parasite, without causing toxicity to the host cell. With the use of flow cytometry, the parasites were analyzed, and it was discovered that cell death was caused by interference with mitochondrial metabolism. As for the effect against the evolutionary forms of *Trypanosoma cruzi*, the paper presented the IC_50_ values for the nanoparticles containing BZN and free BZN. For the epimastigote form, the antiparasitic effects were evaluated at 24, 48, and 72 h. With BZN encapsulated in CaCO_3_ nanoparticles, IC_50_ values of 8.72 μg/mL, 8.02 μg/mL, and 4.8 μg/mL were obtained at 24 h, 48 h, and 72 h, respectively. With the use of free BZN, IC_50_ was 56.7 μg/mL at 24 h, 15.91 μg/mL at 48 h, and 4.3 μg/mL at 72 h. The results show that lower concentrations of the calcium carbonate nanoparticles loaded with BZN were necessary to kill the parasite in a shorter period when compared to free BZN. For the circulating trypomastigotes, the study showed results of the minimum lethal concentration for the parasites (LC_50_), reaching 1.77 ± 0.58 µg/mL with the calcium carbonate nanoparticles loaded with BZN, a value 37 times lower than the one achieved by free BZN, which was 66.9 ± 20.3 µg/mL at 24 h. Circulating trypomastigotes were killed within 24 h at all concentrations tested (50–0.39 μg/mL). However, free BZN was less effective in eliminating the parasite as higher concentrations in the range of 12.5–50 μg/mL had to be administered to reach LC_50_. In tests of inhibition of the intracellular amastigote forms, compound BZN encapsulated in CaCO_3_ nanoparticles was able to reduce by 25% the number of infected cells and by 46% the number of amastigotes per cell with an IC_50_ of 8.72 μg/mL. Free BZN also reduced the number of infected cells by 25% and the number of amastigotes within each cell by 32%, but with a much higher IC_50_ (56.7 μg/mL). Encapsulated BZN showed trypanocidal efficacy against all three forms of *Trypanosoma cruzi* at lower doses when compared to free BZN [22]. 

Another article that showed significant results in in vitro assays was Morales-Baez et al. [23], who synthesized nanoparticles of hybrid materials called MOFs and coupled them to ergosterol peroxide (MOFs-EP), as they have immunosuppressive, antiparasitic, and anti-inflammatory activity. The study analyzed the trypanocidal activity of free MOFs and MOFs coupled to ergosterol peroxide against epimastigotes and trypomastigotes at different concentrations and the toxicity of free MOF nanoparticles by the MTT assay in three cell types. The IC_50_ value of the MOF nanoparticles coupled to ergosterol peroxide was the same for both forms of the *T. cruzi* cycle: 4.81 μg/mL at 24 h and 3.0 μg/mL at 48 h. The application of free MOF nanoparticles at different concentrations and incubation times showed no effect against parasite growth when compared to nifurtimox (positive control), but it was effective when applied in the form of MOF nanoparticles coupled to ergosterol peroxide (MOFs-EP), causing a decrease in parasite growth at different concentrations and incubation times. The cytotoxicity evaluation test by MTT was performed with different concentrations of free MOF nanoparticles applied to cell monolayers in triplicate in three different cell types. The MTT result showed that the free MOF nanoparticles were not cytotoxic when used at different low concentrations; it only showed cell damage when the cells were incubated with 1% Triton X-100 (positive control). The CC_50_ results found for the free MOF nanoparticles were as follows: 392.0 µg/mL for NIH3T3 cells, 593.6 µg/mL for J774A.1 cells, and 1030.0 µg/mL for Vero cells. It was concluded that the MOF nanoparticles did not induce cytotoxicity in cells and may be a promising alternative for the treatment of the acute phase of Chagas disease, due to their selectivity for the plasma membrane, their inhibitory effect against the circulating trypomastigote forms of *T. cruzi*, and for not presenting adverse effects. The study failed to perform tests against the intracellular amastigote form and required complementary testing to assess adverse effects. The selectivity index (SI) was not obtained, and it was not possible to calculate it, as the study did not present CC_50_ results for the MOF nanoparticles containing ergosterol peroxide [23]. 

When comparing both studies, the results obtained by Tessarolo et al. [22] were more complete and had more significant effects. The study managed to obtain 100% of circulating parasites killed within 24 h of treatment with the calcium carbonate nanoparticles loaded with BZN at all administered doses, with an LC_50_ (lethal dose for 50% of the parasites) of 1.77 ± 0.58 µg/mL, lower than the IC_50_ found in the study by Morales-Baez et al. [23]. In addition to that, they carried out tests for all three forms of *T. cruzi*. Although the cytotoxicity of BZN increased when coupled to the nanoparticles, the selectivity index was satisfactory and showed greater selectivity of the calcium carbonate nanoparticles loaded with BZN for the parasite than the free BZN. In the study of Morales-Baez et al. [23], the CC_50_ value was only obtained by the free form of the MOF nanoparticles, and it was not possible to calculate the selectivity index.

#### 3.1.4. Liposomes and Vesicular Nanocarriers

Another study that presented results of in vitro assays was that of Li et al. [1]. The article compared the effects of free BZN with those of the vesicular nanocarrier (poly (ethylene glycol)-block-poly (propylene sulfide) (BZN-PSs)) loaded with BZN. The vesicular nanocarrier (BZN-PSs) was chosen because it had already been used in therapy for effective delivery and reduced toxicity [1]. Trypanocidal efficacy was evaluated in two stages of the *T. cruzi* cycle, against intracellular amastigotes and circulating trypomastigotes. The IC_50_ found for the vesicular nanocarrier loaded with BZN in the effect test against intracellular amastigotes was 3.51 ± 0.79 μM and, for free BZN, it was 33.07 ± 8.17 μM. In assays against circulating trypomastigotes, IC_50_ was 55.87 ± 11.39 μM for the vesicular nanocarrier containing BZN and 56.06 ± 12.21 μM for free BZN. Despite not presenting results on the selectivity index and CC_50_, the study by Li et al. [1] demonstrates, as well as that of Tessarolo et al. [22], significant efficacy in reducing the concentration of the drug against the intracellular amastigote form of the parasite. The IC_50_ result in circulating trypomastigotes was like that of the free BZN found in this study. The article explains that this occurs because the nanostructure manages to take the same route as *T. cruzi* in cell penetration and disintegrate only inside the cell, slowly releasing BZN, which makes it better against intracellular amastigote forms, in addition to contributing to the decrease in toxicity [1]. 

Vinuesa et al. [2] also performed tests with free and BZN-loaded liposomes to assess cytotoxicity in two mammalian cells (L-929 and Hep G2) and the antiparasitic effect against *T. cruzi*. The results found in the cytotoxicity assays were 0 to 39.4 ± 2.4 µg/mL in L-929 fibroblasts and 0 to 36.2 ± 4.9 µg/mL in the Hep G2 cell line for free liposomes. For BZN-loaded liposomes, the results were 0 to 14.9 ± 1.9 µg/mL in L-929 fibroblasts and 0 to 1.5 ± 2.6 µg/mL in Hep G2 cells. The results varied according to the concentrations. Liposome toxicity was considered insignificant in the study. The toxicity result for free BZN in L-929 fibroblast cells was 3.9 ± 4.5 μM and the IC_50_ of the effect against epimastigotes was 14.8 ± 4.9 μM. It was not possible to obtain the IC_50_ of the BZN-loaded liposome, as the BZN concentrations achieved by the liposome were very low, less than 12 μM, rendering its effect against the parasite negligible. The study concluded that liposomes were inefficient in transporting and releasing BZN [2]. 

Among the two articles that used liposomes as BZN carriers in vitro assays, Li et al. [1] managed to achieve better results with the BZN-loaded polymersome vesicular nanocarrier, as it required lower concentrations of encapsulated BZN when compared to its free-form in the effect against intracellular amastigotes. 

Because of all the values presented, the articles that obtained the best results in the in vitro tests were those that worked with inorganic [22], organic [23], and polymeric [4,24] nanocarriers. They showed efficiency in reducing the toxicity and dose of the drug when coupled with nanocarriers. The articles that worked with liposomes and lipid nanocarriers did not obtain satisfactory results [1,2,10].

### 3.2. In Vivo Data Discussion

The discussion was carried out after analyzing the in vivo results of the articles selected in Table 5. Regarding the in vivo tests, the articles were evaluated regarding the results of the tests for reducing animals’ parasite load, survival rate, and mortality. Some papers described tests only in the acute phase of the disease [1,8,27,28,29], and others only did so in the chronic phase [25,26], evaluating the appearance of cardiac and hepatic markers. Other articles obtained results for both phases of the disease [7,30,31]. The nanocarriers used in the in vivo tests were polymeric nanoparticles, lipid nanoparticles, polymersomes, and polymeric micelles. 

#### 3.2.1. Polymeric Nanoparticles

Branquinho et al. [8] used polymeric nanoparticles for treating the acute phase of Chagas disease. Polymeric nanoparticles were synthesized with LYC encapsulated based on PCL and PLA-PEG and their in vivo effects were compared to the drug and to free BZN. Mice were separated into groups for four experiments and infected with different *T. cruzi* strains: CL (sensitive to BZN) and Y (partially resistant to BZN). All administrations were performed intravenously into the rear veins. Free BZN and free LYC were administered in a mixture of DMA-PEG 300 solution diluted in 5% glucose. In all experiments of this study, 2 mg/kg/day doses of free and encapsulated LYC in nanoparticles and 50 mg/kg/day doses of free BZN were administered [8]. In the analysis of the parasitemia peak of the groups infected by the CL strain (sensitive to BZN), in experiment I, initiated 24 h after the infection and lasting 10 days, the result was a reduction in parasitemia by 98.6% with free BZN, by 56.3% with free LYC, and by 96.3% for the LYC-loaded nanoparticles (LYC-PCL NC), when compared to the untreated control groups. As for the survival rate of animals infected by the CL strain (sensitive to BZN), those in the untreated control group survived for approximately 20 days. The groups treated with free BZN and with the LYC-loaded PCL nanoparticles had a 100% survival rate for 6 months after treatment and the animals that received only free LYC had a 75% survival rate in the same period. The remaining 25% survived for 24 days. Experiment II carried out with the CL strain, initiated 7 days after the infection and lasting 20 days, obtained a reduction during the parasitemia peak of 99.2% for free BZN, 57.7% for free LIC, 98.2% for the LYC-loaded PCL nanoparticles, and 99.30% for the LYC-loaded PLA nanoparticles. The results show that free LYC had a lower parasitemia reduction rate than the groups treated with free BZN and with LYC-loaded PLA-PEG and PCL nanoparticles. The survival rate was 21 days for the untreated groups. A total of 50% of the animals treated with free LYC survived for 34 days and 100% of the animals treated with free BZN and with both LYC-loaded nanoparticles survived during the entire acute phase for 6 months after the treatment, until they were necropsied [8].

The animals infected by the Y strain (partially resistant to BZN) were treated in experiment III, initiated 24 h after the infection and lasting 10 days. They obtained peak parasitemia reductions of 94.3% with free BZN, 37.8% with free LYC, and 96.9% by the LYC-loaded PCL nanoparticles when compared to the untreated control group. This experiment did not use the PLA-PEG nanoparticles. Once again, the parasitemia of free LYC remained higher when compared to free BZN and LYC encapsulated in the nanoparticles, although it achieved a greater reduction when compared to the untreated control group [8].

The survival rate was 100% for the groups treated with free BZN and with the LYC-loaded PCL nanoparticles. The animals in the untreated control group survived for only 16 days and treatment with free LYC did not improve survival. In experiment IV, the group began to be treated 4 days after the infection, for a total of 20 days, with PLA-PEG and PLC nanoparticles loaded with LYC and with free BZN, obtaining reductions in the parasitemia peak of 99.55% with the LYC-loaded PCL nanoparticles, of 95.76% for the LYC-loaded PLA-PEG nanoparticles, and of 98.20% for free BZN. The survival rate was the same as in the previous group [8]. 

Treatment efficacy was assessed by parasitological cure. In experiment I, of the animals infected by the CL strain, those treated with free BZN had a 100% cure, while those treated with the LYC-loaded PCL nanoparticles achieved a 50% cure. In experiment II, the animals treated with both nanoparticles and with free BZN had a 100% cure. In both experiments (I and II), none of the animals treated with free LYC or those in the untreated control group were cured. In experiment III, the animals treated with the LYC-loaded PCL nanoparticles achieved a 50% cure, while none of the mice treated with free LYC and free BZN were cured. In experiment IV, a 75% cure was obtained with free BZN, 100% with the LYC-loaded PLA-PEG nanoparticles, and 62.2% with the LYC-loaded PCL nanoparticles. No cure was achieved for the mice in the untreated control group. It is concluded that LYC was able to reduce parasitemia by 56% to 99% and by 37% to 99% in the CL and Y strains, respectively, with intravenous administration of 2 mg/kg/day, depending on the type of formulation and the schedule used [8]. The results could be even better with higher doses. The animals survived without signs of toxicity in the experimental tests with free LYC and with the LYC-loaded PLA-PEG and PCL nanoparticles. Free BZN in a DMA-PEG 300 solution was able to cure 75% of the animals with a 50 mg/kg/day dose, a better result than the one found with the standard dose of 100 mg/kg/day used for treating Chagas disease, which reached a 50% healing rate [8].

Despite the reduction in parasitemia and the survival rate of mice infected with the CL strain, experiment I achieved a cure rate of 50% using the LYC-loaded PCL nanoparticles, not outdoing free BZN, which reached 100%. The study explains that this result could be due to the high sensitivity of the CL strain to BZN and to the short treatment period, which was initiated 24 h after the infection and lasted only 10 days. The results of experiment II were better, achieving total survival and complete cure with the LYC-loaded PLA-PEG nanoparticles, with the LYC-loaded PCL nanoparticles, and with free BZN. The treatment in experiment II was initiated 7 days after the infection and lasted 20 days. It is believed that the results were better than experiment I because, after this period, the intracellular amastigote forms had already multiplied and ruptured the host cells, releasing the circulating trypomastigote form in the bloodstream, encountering the drugs and with the nanoparticles [8]. The same occurred in the experiments with the Y strain, partially resistant to BZN. The action of encapsulated LYC was better than that of free LYC due to the ability of the nanoparticles to carry out a prolonged release of the drug. As was the case in experiment I, experiment III carried out the treatment for 10 days, starting it 24 h after the infection, which resulted in a reduction in parasitemia and cure in 50% of the animals only with the use of the LYC-loaded PCL nanoparticles. Once again, the above shows the importance of longer treatment and waiting times to start after the infection. Experiment IV obtained a 100% parasitological cure with the LYC-loaded PLA-PEG nanoparticles, 75% with free BZN, and 62.5% with the LYC-loaded PCL nanoparticles. Free LYC ceased to be used in this experiment due to its lower results to the nanoparticles. In the entire treatment, 50% of the animals treated with free LYC did not survive and did not present a parasitological cure. The above shows the importance of encapsulation in nanocarriers [8].

De Mello et al. [30] also used PLA-PEG and PCL polymeric nanoparticles containing the LYC-encapsulated natural active ingredient for in vivo assays. The nanoparticles were prepared by the technique of interfacial deposition of the preformed polymer followed by solvent displacement. The paper carried out tests during the acute and chronic phases of the disease in mice infected with the *T. cruzi* Y strain (partially resistant to BZN) and compared the results. The animals were infected intraperitoneally with 10,000 circulating trypomastigotes for the acute phase and 500 circulating trypomastigotes for the chronic phase. In the acute phase, the animals were orally treated with both LYC-loaded nanoparticles and with free LYC at a dose of 5 mg/kg/day for 20 days, starting on the 4th day after the infection, as was performed in experiment IV of the article by Branquinho [8]. In the chronic phase, free LYC and both LYC-loaded nanoparticles were administered orally (5 mg/kg/day) and intravenously (2 mg/kg/day) for 20 days, starting 90 days after the infection. For comparison purposes, two groups of animals were treated with 100 mg/kg/day of free BZN orally and 50 mg/kg/day intravenously [30].

Fresh blood tests, PCR, blood culture, and enzyme-linked immunosorbent assay (ELISA) were performed to assess therapeutic efficacy. The cure rate achieved with the use of the LYC-loaded PLA-PEG nanoparticles was 62.5% in the acute phase by the oral route, 55.6% in the chronic phase by the oral route, and 50% in the intravenous route. With the LYC-loaded PCL nanoparticles, the cure rates were 57.0% in the acute phase by the oral route, 30.0% in the chronic phase by the oral route, and 33.3% by the intravenous route. As in the results obtained by Branquinho et al. [8], no animal was cured with the use of free LYC, despite managing to reduce parasitemia and increase the survival rate when compared to the control group without treatment. The animals treated with free BZN achieved a cure rate like those treated with nanoparticles during the acute phase. In the chronic phase, only the animals treated with the LYC-loaded nanoparticles were cured, both in oral and intravenous administration. Treatment with the LYC-loaded PLA-PEG nanoparticles and free BZN achieved total parasitemia suppression in the acute phase. The survival rate of the animals was 75% with free LYC, 87% with the LYC-loaded PCL nanoparticles, and 100% with the LYC-loaded PLA-PEG nanoparticles up to 6 months after treatment when they were necropsied. In the control groups, the animals survived for approximately 20 days [30].

The survival rate in the chronic phase was 70% (orally) and 40% (i.v.) with free LYC, 80% (orally) and 50% (i.v.) with free BZN, 100% (orally) and 90% (via i.v.) with the LYC-loaded PCL nanoparticles, and 90% (orally) and 100% (via i.v.) with the LYC-loaded PLA-PEG nanoparticles. The survival rate of the untreated groups was 70% by the oral route. The results demonstrated greater efficacy of the LYC-loaded PLA-PEG nanoparticles in the treatment of the disease when compared to free LYC. The higher efficiency of nanoparticles containing LYC against the parasite is due to the controlled release of the active drug, as it has a smaller particle size, the ability to stabilize the drug in the gastrointestinal tract, and to influence tissue diffusion and LYC uptake into the blood [30]. 

Despite having good permeability, rapid absorption, and distribution in its free form, LYC has poor solubility. In addition to that, it requires the administration of repeated doses due to its rapid elimination. In oral administration, it shows chemical instability in contact with the pH of the gastrointestinal tract [30]. Encapsulation in polymeric nanoparticles controls the drug release in a slow and sustained way, which improves pharmacokinetics and biodistribution; in addition to reducing the need for multiple doses, which consequently reduces toxicity. Polymeric nanoparticles are more stable than other nanocarriers, such as lipid-based ones, liposomes, and micelles. The polymeric wall stabilizes the encapsulated drug and increases circulation time by reducing opsonization and release by the phagocytic system. Degradation of the polymeric wall and the release of LYC to the external environment are reduced by preventing the entry of proteins into the oil core. As they do not have elimination and accelerated metabolism like free LYC, LYC-loaded polymeric nanoparticles manage to accumulate in regions of inflamed tissues, improving their activity and being more effective [30]. Although the results achieved by the nanoparticles are close to those found with free BZN, it is important to emphasize that this shows the significant efficacy of these nanocarriers, as they do not compromise the action of the drugs but achieve excellent results by reducing the dose required for the treatment, which is essential to obtain the lowest possible level of adverse effects. In addition to that, the aqueous suspension of LYC-encapsulated nanoparticles is a physiologically acceptable vehicle for oral administration. Having achieved a cure in the chronic phase by this study is encouraging, as it is a phase of the disease with virtually no effective conventional treatments. 

Branquinho et al. [31] also evaluated in vivo assays of the action of the PLA-PEG polymeric nanoparticles loaded with LYC. Treatment was performed by administering higher doses orally to mice infected with a strain (VL-10) resistant to BZN and nifurtimox during the acute and chronic phases of the disease. During the acute phase, treatment was initiated 9 days after the infection and lasted 20 days. In the chronic phase, it was initiated 90 days after the infection, with the same duration. A total of 12 mg/kg/day of free LYC, 8 or 12 mg/kg/day of LYC-loaded PLA-PEG nanoparticles, or 100 mg/kg/day of free BZN were administered. With the tests in the acute phase of the disease, the LYC-loaded PLA-PEG nanoparticles managed to suppress parasitemia by 100% with a 12 mg/kg/day dose and by 50% with the lowest dose: 8 mg/kg/day. At a dose of 12 mg/kg/day of the LYC-loaded PLA-PEG nanoparticles, the results obtained were 75% cure in the acute phase and 88% cure in the chronic phase of the disease. At a dose of 8 mg/kg/day, the cure was 43% both in the acute and chronic phases. The animals treated with free BZN and free LYC were not cured, as was also the case with untreated controls. In all groups, the survival rate was 87.5% in the acute phase and, with the LYC-loaded PLA-PEG nanoparticles, it was 100%. In the chronic phase, the survival rate with the use of 12 mg/kg/day of the nanoparticles or with 100 mg/kg/day of free BZN was 80%, while with the dose of 8 mg/kg/day of the nanoparticles and free LYC, it was 70% [31].

In all three articles discussed, the treatment with polymeric nanoparticles showed better results when compared to those achieved by the drugs in their free form. The result varies according to the *T. cruzi* strain, the dose used in the treatment, the length of the treatment time, and the start date. The article [8] tested a 2 mg/kg/day dose during treatment (10 and 20 days) and different strains in the acute phase. The results after 20 days were better, as well as in the studies by De Mello et al. [30] and Branquinho et al. [31]. The treatment was also more effective when initiated 4 to 9 days after the infection in the acute phase, as shown in experiments II and IV by Branquinho et al. [8] and in the tests by De Mello et al. [30] and Branquinho et al. [31]. Free LYC failed to cure any of the animals, while free BZN did not cure mice infected with the BZN-resistant VL-10 strain [31] but achieved high cure rates against sensitive (CL) [8] and partially resistant strains to BZN (Y) [30]. LYC-loaded polymeric nanoparticles achieved high cure rates against all strains at lower doses than those administered with free BZN.

The results of the tests carried out in the chronic phase of the disease were encouraging. The article [30] obtained a cure rate of approximately 55% with the LYC-loaded PLA-PEG nanoparticles and approximately 33% with the LYC-loaded PCL nanoparticles. There was no significant difference between the results with oral and intravenous administration. The study by Branquinho et al. [31] obtained a higher cure rate (88%) of mice in tests in the chronic phase with the administration of a higher dose: 12 mg/kg/day. In both studies, free BZN and free LYC failed to cure any animals in the chronic phase tests. In conclusion, the studies showed that LYC encapsulated in polymeric nanoparticles was more effective in reducing parasitemia and in achieving a parasitological cure, and increased animal survival, even when administered at low doses (2 mg/kg/day), when compared to free BZN (100 to 50 mg/kg/day). Encapsulated LYC presented no toxicity in any of all three studies, thus representing a promising alternative for the treatment of Chagas disease.

#### 3.2.2. Lipid Nanoparticles

In addition to the aforementioned in vitro assays, the study by Carneiro et al. [10] carried out in vivo assays comparing the action against the circulating trypomastigote form of *T. cruzi* of the solid lipid nanoparticles loaded with compound H2bdtc (5-hydroxy-3-methyl-5- phenyl-pyrazoline-1-(S-benzyl dithiocarbonate) to its free form and to free BZN. The tests were performed on mice infected with the *T. cruzi* Y strain. The treatment was administered orally with 1.0 mg/kg/day of free BZN (100 times less than the standard concentration for the treatment of Chagas disease) and 1.4 mg/kg/day of free H2bdtc and H2bdtc-encapsulated in SLNs, for 10 consecutive days. The treatment was initiated on the fifth day after the infection, which reached its peak 9 days later. The results showed a 70% reduction in parasitemia with the administration of the 2bdtc-loaded solid lipid nanoparticle, 45% with free H2bdtc, and 15% with free BZN (positive control) when compared to the control group. The survival rate of the mice was 100% using the 2bdtc-loaded solid lipid nanoparticles. This rate is comparable to the one obtained with the standard administration of free BZN at 400 μmol/kg/day, 100 times higher than the one used in this study with the nanoparticles. With free BZN and H2bdtc, the survival rate compared to the control group was 57%. The study also evaluated whether there was heart and liver damage in the animals after the treatment [10]. The results showed that the animals treated with the nanoparticles did not show cardiac lesions, only reduced inflammation. The H2bdtc compound in its free form reduced cardiac damage by 50% when compared to free BZN in the standard treatment. The H2bdtc-loaded in SLNs also reduced liver toxicity and liver inflammation caused by the *T. cruzi* infection. Both in the in vitro and in vivo assays, the encapsulation of the H2bdtc compound obtained better results than those found with free BZN when administered at the same concentrations as in the study. The article shows that the nanoparticles were able to increase the oral bioavailability of the drug and improve solubility, which may facilitate access to the parasite. The study concludes that the compound is more efficient than the drugs currently used against the *T. cruzi* infection [10].

#### 3.2.3. Polymeric Micelles

The articles [26,27,28] studied BZN-loaded polymeric micelles (BZN-PMs). Rial et al. [27] and Scalise et al. [28] performed in vivo assays during the acute phase of the disease.

Rial et al. [27] administered polymeric micelles dispersed in olive oil by oral gavage at doses of 10, 25, and 50 mg/kg/day in different groups of mice, for 30 days, starting two days after infection, and compared the results obtained to free BZN dispersed in olive oil at a dose of 50 mg/kg/day by oral gavage. All mice treated with free BZN and with the BZN-loaded polymeric micelle survived until the end of the treatment. In the control group, only 15% of the animals survived. The saturation solubility of BZN encapsulated in polymeric micelles had a 10-fold increase (3.99 mg/mL) when compared to the solubility of free BZN (0.44 mg/mL) [27]. Improved solubility and absorption of BZN encapsulated in polymeric micelles (BZN-PMs) provided an increase in the animals’ survival rate with a 10 mg/kg/day dose, much lower than the conventional 100 mg/kg/day free BZN dose. The decrease in the dose achieved by encapsulating BZN in polymeric micelles with sustained release improves the pharmacological action, can avoid possible adverse effects, and reduces the administration frequency [27]. 

Scalise et al. [28] carried out tests for 15 and 30 days with mice separated into groups, administering oral doses of 50, 25, and 10 mg/kg/day with BZN-loaded polymeric micelles (BZN-PMs). At doses of 50 and 25 mg/kg/day, all mice survived for at least 50 days. Mice infected and treated with a 10 mg/kg/day dose had a survival rate of 70%. Thus, it can be stated that the efficacy of the treatment is dose dependent. Furthermore, as in the study by Rial [27], BZN-loaded polymeric micelles had a significant antiparasitic effect and prevention of animal death even at lower doses. 

Rial et al. [26] used BZN-loaded polymeric micelles (BZN-PMs) in 1-month-old female C57BL/6J model mice of similar weight infected with the Nicaragua strain of *T. cruzi* during the chronic phase of the disease. The treatment was carried out by separating the animals into different groups and comparing the efficacy of administering BZN-MP continuously with intermittently. For the continuous treatment, the animals received BZN-PM doses of 25 and 50 mg/kg/day for 30 days. For the intermittent treatment, they received 13 doses of 50 and 75 mg/kg every 7 days. The administration was via the oral route. The results showed that the intermittent doses were as effective as those administered continuously, which is beneficial because the total intermittent dose is smaller. All animals survived until the end of treatment. The evaluation of the efficacy of the treatment by qPCR showed that 80% of the animals continuously treated with a 50 mg/kg/day dose had negative results. All other groups treated with BZN-PMs had no parasite load detected. The authors compared the results with previous studies and revealed that 80% of the animals treated with continuous doses of 75 mg/kg did not present parasitemia, while for the group treated with intermittent doses of 100 mg/kg, 75% did not present parasitemia [26]. 

The study also evaluated levels of *T. cruzi*-specific antibodies. The results showed a reduction in the antibody levels in all treatment groups when compared to the untreated ones, as well as a better reduction with the intermittent BZN-PM administration. Cardiac electrical alterations in mice were evaluated by ECG, considering that the conduction alterations caused by the infection were reverted after BZN-PM administration continuously and intermittently. Despite this, free BZN showed the same efficacy as polymeric micelles. The study also provoked immunosuppression of the animals after the treatment to evaluate the reactivation of the parasite, not detecting blood parasitic load [26].

When compared to previous studies carried out by Rial et al. [26], a significant reduction was obtained concerning the standard dose of 100 mg/kg/day, being equally effective with a dose of 75 mg/kg/day. This equated to an approximately 70% reduction in the total dose. This improvement is due to the BZN encapsulation in micelles, which are smaller than standard BZN and increase solubility from 0.4 mg/mL to 3.99 mg/mL [26]. Good results were obtained in the administration of smaller BZN-PM doses, as shown in this study with intermittent doses, as they can be an alternative to long-term treatments with fewer adverse effects on the patient. 

#### 3.2.4. Liposomes

In addition to the tests performed in vitro, the article [1] also evaluated the action of the vesicular nanocarrier (polymersome) with encapsulated BZN (poly (ethylene glycol)—block—poly (propylene sulfide) (BZN-PSs)) in in vitro assays in BALB/c mice infected with the *T. cruzi* Y strain, during the chronic phase of Chagas disease. The animals were separated into groups and the treatment was initiated 7 days after the infection with the administration of three different concentrations of the BZN-loaded vesicular nanocarrier (0.03, 0.15, and 1.5 mg/kg), 100 mg/kg for the group treated with free BZN, empty nanocarrier, and an untreated group. The results showed that the BZN-loaded vesicular nanocarrier obtained better reduction rates in cardiac parasitemia and myocarditis at a concentration of 1.5 mg/kg. The lowest dose of 0.03 mg/kg reduced parasitemia by 50%, while the mean dose of 0.15 mg/kg failed to reduce cardiac parasitemia but did reduce myocarditis (heart inflammation). At its standard treatment dose (100 mg/kg/day), free BZN was less effective in reducing parasitemia when compared to doses of the BZN-loaded vesicular nanocarrier. In addition to that, no hepatotoxicity was detected in mice using BZN-PS. The amount of BZN present in both nanocarriers administered during all 14 days of the treatment was 466 times lower than the one normally used with free BZN, achieving similar results in reducing parasitemia. The most important result achieved by the study was the reduction in myocarditis/cardiomyopathy (heart inflammation), the most serious sequel caused by infection with the parasite. Once again, using the encapsulated form of drugs in nanocarriers demonstrates significant efficacy in reducing the necessary doses for the treatment against the *T. cruzi* parasite, without causing undesirable adverse effects to the patients. Despite being promising, nanocarriers still need to be tested in clinical studies to evaluate their action in humans [1].

After analysis of the articles discussed, it is concluded that the best results were obtained with the use of drugs encapsulated in polymeric nanoparticles, as shown in the studies by Branquinho et al. [8], De Mello et al. [30], and Branquinho et al. [31]. The best length of the treatment in time was 20 days, starting 4 to 7 days after the infection. The studies analyzed the oral and intravenous routes, not showing significant differences in the results, with oral administration as the best choice for the patients’ adaptation. In addition to BZN, the first-choice drug used against the *T. cruzi* parasite, LYC is a natural product encapsulated in polymeric nanoparticles that proved to be a great alternative for the treatment, obtaining high parasitemia reduction rates, as well as increased survival and cure rates for the animals during the acute and chronic phases of the disease, with doses much lower than those used in standard treatments with free BZN. Their studies can be improved and progress to future clinical trials. 

Studies have shown that nanocarriers offer several advantages over traditional drugs for the treatment of Chagas disease. One of these advantages is that nanocarriers can improve the bioavailability of the drug, allowing it to reach specific sites more efficiently, improving the therapeutic index [13]. Nanocarriers administered orally need to be absorbed in the intestine by permeation for drug release into the blood or for cellular uptake. Furthermore, nanocarriers can slowly release the loaded actives which can reduce blood peaks of the drug and consequently adverse effects [1,23,24].

Nanocarriers can stabilize the active in the gastrointestinal tract and can promote the tissue diffusion and drug uptake into the blood [14,22,30]. Nanocarriers can also increase cellular uptake of the drug (Figure 2), which can kill the parasite in a shorter time compared to the free drug. In addition, nanosystems can accumulate in inflamed tissues, improving action and effectiveness. The use of nanocarriers for the treatment of Chagas disease also has some disadvantages, such as the difficulty in large-scale production, since the production of nanocarriers requires complex processes, which can increase the cost of treatment. In addition, toxicological and genotoxic studies must be performed to investigate the risk of human exposure to nanocarriers. The results showed that nanocarriers are also dose dependent and their effect varies according to the sensitivity of the *T. cruzi* strain in relation to the drug [28,30]. The advantages of nanocarriers as drug delivery systems are real, but there are many challenges to be overcome. It is necessary to evaluate the safety and efficacy of treatments using nanocarriers and to develop methods to produce them on an industrial scale to promote clinical studies.

#### 3.2.5. Nanocarriers of Drugs in the Chronic Phase of Chagas Disease

Chagas disease has two phases: acute and chronic. The acute phase exhibits high numbers of trypomastigotes in the blood and is controlled by the immune system with the help of drugs. After the acute phase, the parasite continues the life cycle being present outside and inside host cells. In the chronic phase, parasitemia in the blood is low and detection is difficult. In addition, the symptoms are not felt for years, but 4 out of 10 patients develop the chronic phase of Chagas disease. Intracellular amastigotes promote tissue damage to the heart, esophagus, and colon. Patients die due to heart failure caused by chronic chagasic cardiomyopathy [37,38].

There are two strategies for targeting nanocarriers to the target site: passive and active targeting. Both strategies work well for treating tumors and inflamed tissues. Cancer treatments involving nanocarriers depend on the enhanced permeability and retention effect (EPR effect). Tumor tissue grows quickly and releases a series of substances capable of stimulating neoangiogenesis. The vessels grow rapidly in a defective way and present defects such as holes allowing the passage of nanocarriers from the blood vessel into the tissue. Nanocarriers in the tissue interstitium can be endocytosed into the cytoplasm of cells. In active targeting, nanocarriers decorated with targeting molecules pass through the holes in the vessels and can bind to cells, facilitating the uptake. Inflamed tissues have permeable blood vessels, and this fact can be used in favor of the passive or active targeting of nanocarriers [37,39,40]. The use of nanocarriers to target tissue is a success in the treatment of tumors and inflammation; however, targeting these delivery systems to cardiac muscle in the chronic phase of Chagas disease is a challenge. Nanocarriers are effective in transporting drugs to cardiomyocytes when these systems escape from blood vessels into the interstitium of cardiac tissue. In the interstitium, nanocarriers can be recognized and taken up by cardiomyocytes by the mechanism of endocytosis, which is a route to reach the amastigotes. However, the number of amastigotes in cardiac tissue is small and the tissue lesions are slow. Thus, for every significant piece of damage to the cardiac tissue caused by amastigotes, local inflammation is established. Initially, inflamed tissue can facilitate the passage of the nanocarriers to the interstitium, but at the end of the inflammatory process there is a reduction in local circulation and hypoxia [37]. Thus, there is probability of success for the nanocarriers to reach cardiomyocytes at the beginning of the chronic phase or indeterminate phase where blood flow has not yet been compromised. In the late chronic phase, cardiac tissue has suffered extensive damage, compromising the blood flow and the effectiveness of nanocarriers.

There are articles published with promising results from the use of drugs encapsulated in nanocarriers for the treatment of Chagas disease in the chronic phase [7,25,26,30,31]. The articles use animal models and, probably, drugs encapsulated in nanocarriers are effective at the beginning of the chronic phase or indeterminate phase, where circulation in the cardiac tissue has not been compromised, with the possibility of escape of the nanocarriers from the blood vessels to the cardiac tissue.

## 4. Materials and Methods

To prepare this paper, a search of the literature was carried out in the PubMed and Web of Science scientific databases from January 2012 to May 2023, using the following combinations of keywords: “Chagas Disease and nanoparticles”, “*Trypanosoma cruzi* and nanoparticles” and “Chagas Disease and *Trypanosoma cruzi* and nanoparticles”. Subsequently, the articles found were assessed using the inclusion and exclusion criteria defined for this review. For the inclusion criteria, the articles considered were those that used nanocarriers in in vitro and in vivo studies in the search for new ways to improve the treatment of Chagas disease. According to the exclusion criteria established, review articles, patents, duplicates, book chapters, conference abstracts, articles published in journals with an impact factor less than 1.0, articles on vaccines, and articles that deviated from the topic proposed were excluded. The scoping review was conducted by three individuals to ensure the quality of study selection. For a better detailing of the search, specific spreadsheets were prepared using Microsoft Excel (2019) for the in vitro and in vivo assays, for the composition and physicochemical characterization of the nanostructured systems, as well as a final evaluation table for each article, regarding the presence of certain items, such as title appropriate to the content, abstract, contextualization and objective present in the introduction, ethical statement, study design, complete physicochemical characterization, size of the nanocarriers, animals, sample size, in vitro and in vivo studies, trypanocidal effect, statistics, interpretation of the results, limitations, and conclusion.

## 5. Conclusions

After a detailed analysis, this study showed that using nanocarriers capable of encapsulating drugs achieved efficient results in the treatment of the disease. In in vitro assays, the encapsulated drugs did not lose their trypanocidal activities against the different forms of *T. cruzi*. In the tests employed, they were able to reduce cellular toxicity and increase the selectivity of the drugs against the parasite. With the in vivo studies, carried out with mice, the tests in both phases of the disease (acute and chronic) showed a reduction in parasitemia and a high survival and cure rate in the animals with the administration of much smaller doses when compared to the standard treatment with free BZN. In addition to healing during the chronic phase, some results with drugs encapsulated in nanocarriers have shown a reduction in liver toxicity and heart inflammation, which is encouraging given the lack of treatments available for this clinical condition.

With the increased circulation of drugs provided by the prolonged release and the possible decrease in adverse effects by reducing the necessary doses, the nanocarriers showed physiological stability, efficacy, and safety in transporting and delivering drugs to the target. 

The nanotechnological strategies for drug delivery could open a new horizon of clinical practices for the successful treatment of Chagas disease. One of the strategies, nanoparticles containing drugs, has shown promising results in inducing the host’s immune response against the pathogen with few side effects; in addition, it showed promising results in reducing toxicity, elevating efficacy, and the bioavailability of the active compound against the pathogen, by prolonging release, thereby increasing the therapeutic index. However, there is a need for more clinical studies to predict whether the nanoparticles will offer improved effective antichagasic treatments since the benefits of the nanotechnology for this disease are excellent in relation to cellular assays which have stimulated the preliminary animal studies [6,13,37]. Moreover, the unique nanocarrier approved for clinical trials is liposomal amphotericin B (LAMB) with reasonable anti-*T*. *cruzi* activity. From this perspective, the pre-clinical studies of the nanocarriers containing anti-*T*. *cruzi* drugs should be encountered, and this situation demands an approach from governmental and private agencies in conjunction with the pharmaceutical industry focusing on the efficient implementation of these nanosystems for drug delivery that will elevate the efficacy of anti-chagasic treatment procedures [6,13].

The limitations of nanocarriers are related to their ability to deliver drugs to tissues with low blood flow during the chronic phase of the disease. The targeting of nanocarriers to cardiac muscle in the chronic phase of Chagas disease is a challenge due to tissue fibrosis and low blood flow.

The results of preclinical studies analyzed by this review were important in the search for new alternatives for the treatment of Chagas disease. More studies can be carried out so that the treatment is perfected and can proceed to future clinical studies that may provide better adherence by the patients.

## Figures and Tables

**Table 1:** Keyword research and number of articles found.

Keywords	PubMed	Web of Science	Total
Chagas disease and nanoparticles	69	63	123
*Trypanosoma cruzi* and nanoparticles	49	71	120
Chagas disease and *Trypanosoma cruzi* and nanoparticles	43	44	87

**Table 2:** Evaluation of the articles selected, according to the binary code 0/1, where 0 = not present and 1 = present. The value 0 indicates not present and value 1 indicates present. Table adapted from Cruz [21].

Reference	[22]	[23]	[24]	[2]	[25]	[4]	[3]	[10]	[1]	[26]	[5]	[27]	[28]	[29]	[8]	[30]	[31]	[7]
Title	1	1	1	1	1	1	1	1	1	1	1	1	1	0	1	1	1	1
Abstract	1	1	1	1	1	1	1	1	1	1	1	1	1	1	1	1	1	1
**Introduction**																		
Contextualization	1	1	1	1	1	1	1	1	1	1	1	1	1	1	1	1	1	1
Objective	1	1	1	1	1	1	1	1	1	1	1	1	1	1	1	1	1	1
**Methods**																		
Ethics statement	0	0	0	0	1	0	0	1	1	1	0	1	1	1	1	1	1	1
Study design	0	0	0	0	1	0	0	1	1	1	0	1	1	1	1	1	1	1
Complete physicochemical characterization	0	0	1	1	0	0	0	1	1	0	1	0	0	1	0	0	1	0
Nanometric size	1	1	1	1	1	1	0	1	1	1	1	1	1	1	1	1	1	1
Animals	0	0	0	0	1	0	0	1	1	1	0	1	1	1	1	1	1	1
Sample size	0	0	0	0	1	0	0	1	1	1	0	1	1	1	1	1	1	1
In vitro study	1	1	1	1	0	1	1	1	1	0	1	1	1	1	0	0	0	1
In vivo study	0	0	0	0	1	0	0	1	1	1	0	1	1	1	1	1	1	1
Trypanocidal effect	1	1	1	0	1	1	1	1	1	1	1	1	1	1	1	1	1	1
Statistics	1	1	1	1	1	1	0	1	1	1	1	1	1	1	1	1	1	1
**Results and discussion**																		
Interpretation	1	1	1	1	1	1	1	1	1	1	1	1	1	1	1	1	1	1
Limitations	1	0	1	0	1	1	1	1	1	1	1	1	1	1	0	1	1	1
Conclusion	1	1	1	1	1	1	1	1	1	1	1	1	1	1	1	1	1	1
**Score**	**11**	**10**	**12**	**10**	**14**	**12**	**9**	**17**	**17**	**15**	**12**	**16**	**16**	**16**	**14**	**15**	**16**	**16**

**Table 3:** Composition and physicochemical characterization of the Nanocarriers.

**Reference**	**System**	**Composition**	**Drug**	**Drug Concentration**	**Preparation Method**	**Characterization**	**Size (nm)/PDI**
**Polymeric Nanoparticle**							
[4]	Chitosan polymeric nanoparticles (CS-NPs) and S-nitroso-MSA-CS NPs (NO-releasing nanoparticles)	CS, TPP, MSA, NaNO_2_, DTNB, EDTA, PBS, [3-(4,5-dimethylthiazol-2-yl)-2,5-diphenyl-2H-tetrazolium bromide] and acetic acid	Mercaptosuccinic acid (MSA) and NO for NaNO_2_	400 mmol/L of MSA and equimolar amount of NaNO_2_	Ionotropic gelation, stirring and suspension	Mean hydrodynamic diameter, PDI, and zeta potential by DLS. MSA EE in CS-NPs and nitrosation of MSA-CS NPs by UV-Vis spectrophotometry. Kinetics of decomposition through linear regression.	101.0 ± 2.535 nm/ PDI = 0.280 ± 0.006
[7]	Biodegradable polymeric nanoparticles (NPs) with encapsulated LYC	LYC, monomethoxy-polyethylene glycol-block-poly(lactide) polymer, n-dimethylacetamide: polyethylene glycol 300 (DMA-PEG), isotonic glucose solution	LYC	10 mg of LYC with 60 mg of PLA.	Interfacial polymer deposition followed by solvent displacement method.	Mean hydrodynamic diameter and PDI determined by DLS	105.1 ± 4.4 nm/ PDI = Below 0.3
[8]	LYC-loaded polymeric nanoparticles	LYC, DMA-PEG 300, Glucose, Acetone, Poloxamer 188, PLA-PEG Polymer, Resomer 203, Epikuron 170, Miglyol 810 N	LYC	10 mg de LYC with 80 mg PCL Drug loading 95% to LYC-loaded in PCL-NP 10 mg de LYC with 80 mg (40 mg of PLA-Peg + 40 mg Resomer 203) Drug loading 100% to LYC-loaded in (PLA-Peg/PLA)-NP	Interfacial polymer deposition followed by solvent displacement method.	Mean size and PDI	182.5 ± 3.2 nm/ PDI = Below 0.3
[24]	Polymeric PLA nanoparticles containing DETC	DETC, acetone, ethanol, and PLA at 0.5% (*w*/*v*)	Diethyldithio-carbamate	1:12 DETC/polymer ratios, which corresponds to 8.2% (*w*/*w*) of the drug in the system	Nanoprecipitation with solvent evaporation methodology	Physical characterization by DLS, SEM, and AFM. Particle diameter by DLS, zeta potential by electrophoretic mobility. EE, DL, UV-Vis spectrophotometry, and FTIR-ATR	168 nm/ PDI below 0.3
[25]	Polymeric nanoparticle	PLGA, curcumin, ethyl acetate, polyvinyl alcohol, 5% sucrose solution. (coating), miltefosine (co-release vehicle)	BZN and Curcumin	500 mg of PLGA with 75 mg of Cur	Emulsification followed by evaporation	DLS size, zeta potential, and AFM morphology	250–300 nm
[29]	Polymeric PCL nanoparticles	UA, PCL, acetone, and surfactant (Poloxamer™ 407).	Ursolic acid	125 mg of PCL with 12.5 mg of ursolic acid	Nanoprecipitation	Size, zeta potential, PDI, EE, morphology by SEM, and thermal behavior by DSC	1:1 = 197.6 ± 0.85 nm 1:2 = 173.2 ± 7.28 nm/ PDI = 0.09 ± 0.03
[30]	Polymeric PEG nanoparticles containing LYC (LYC-NPs)	LYC, PCL, Miglyol 810 N, Epikuron, Acetone, Poloxamer 188, PLA-PEG and PLA	LYC	20 mg of LYC with 80 mg of PCL. 20 mg of LYC with 60 mg of PLA-PEG diblock polymer blended with 60 mg of PLA homopolymer.	Interfacial polymer deposition followed by solvent displacement method.	Mean hydrodynamic diameter and PDI by DLS. Zeta potentials by laser Doppler anemometry associated with microelectrophoresis	LYC-PCL-NP 190.2 ± 5.7 nm LYC-PLA-PEG-NP 106.1 ± 6.3 nm/ PDI below 0.3
[31]	Lychnopholide Polymeric Nanoparticles (LYC-PLA-PEG-NP)	LYC, PLA-PEG. Acetone, Epikuron, Miglyol, Poloxamer	LYC	20 to 40 mg de LYC with 120 mg (60 mg of PLA-Peg + 60 mg Resomer 203) LYC loading in PLA-PEG-NP of 9 wt%	Interfacial polymer deposition followed by solvent displacement method.	Mean hydrodynamic diameter, PDI, zeta potential, HPLC-UV, AFM, and LYC loading.	107 ± 8 nm PDI = Below 0.3
**Lipid Nanoparticles**							
[2]	SLNs, NLCs and liposomes.	BNZ, Precirol^®^ dichloromethane solution, Poloxamer 188 at 1% and Polysorbate 80 for SLNs. Precirol^®^ ATO 5, Miglyol^®^ 812, Polysorbate 80 and Poloxamer 188 for NLCs. Cholesterol with PEG1000 for Liposomes.	BZN	-	Emulsification techniques for SLNs. Hot homogenization technique using high pressure homogenizer for NLCs. Fluid compression technique called DELOS-SUSP for liposomes.	Size, PDI, Zeta potential, EE, and cumulative release	SLNs (166 nm) NLCs (202 nm) Liposomes (118 nm) PDI SLNs (0.219 ± 0.02–0.263 ± 0.02) NLCs = 0.371 ± 0.03–0.447 ± 0.00) Liposomes (0.190 ± 0.005)
[10]	H2bdtc-loaded SLNs (H2bdtc-SLNs)	Sodium taurodeoxycholate, melted stearic acid, soy lecithin and H2bdtc	H2bdtc	0.02% *w*/*v* of H2bdtc with 0.12% *w*/*v* of sodium taurodeoxycholate and 0.95% *w*/*v* of stearic acid	Microemulsion	D-stroke size by PCS. Zeta potential by mobility electrophoresis of the nanoparticles. Morphology by AFM. DL by UV-Vis spectroscopy. EE%	127.4 ± 10.2 nm/ PDI = 0.229 ± 0.130
**Mesoporous Silica Nanoparticles**							
[3]	Mesoporous Silica Nanoparticles (MSNs)	BZN, CS, EtOH, GPTMS, NaOH, HCl, C_4_H_4_O_3_, TEOS, CTAB.	BZN	-	Positively charged CTAB model and NaOH catalyst in diluted aqueous conditions, through hydrolysis and condensation of tetraethoxysilane	Zeta potential, TEM, EFTEM, CHN, XPS, SS NMR and DFT	Not reported
**Silver Nanoparticles**							
[5]	Silver nanoparticles	*Iresine. herbstii* leaves, corn cob xylan, silver nitrate	Xylan (bioactive polysaccharide)	10 mg/mL (1:9 *w*/*v*) solution of xylan with a solution of 1.0 mM silver nitrate.	Green Synthesis process, continuous agitation, centrifugation and lyophilization	Ultraviolet-visible spectroscopy, FTIR, Raman spectroscopy, EDS, SEM, AFM, ICP-OES, DLS, PDI, and zeta potential	55 nm mean size by SEM and AFM DLS showed 102 ± 1.7 nm PDI = 0.178
**Polymeric Micelles**							
[26]	Polymeric micelles (BZN-PMs)	BZN, ethanol and Lutrol F-68 (P188)	BZN	200 mg of BZN dissolved in 10 mL of EtOH and 300 mg of P188	Solvent diffusion method	Size, zeta potential, and PDI	61–65 nm/ PDI = 3.35 ±0.1
[27]	BNZ polymeric micelles (BZN-PMs)	BZN, EtOH (solvent), water (antisolvent), Lutrol F-68 (P188)	BZN	200 mg of BZN dissolved in 10 mL of EtOH and 300 mg of P188	Solvent diffusion method and nanoprecipitation technique with P188 as stabilizer	Size by DLS, PDI, ID, z mean diameter, and zeta potential (ζ). Saturation solubility studies	63.30 ± 2.82 nm/ PDI = 3.35 ± 0.10
[28]	Polymeric micelles (BZN-PMs)	BNZ, EtOH and Lutrol F-68 (P188)	BZN	200 mg of BZN dissolved in 10 mL of EtOH and 300 mg of P188	Solvent diffusion method and nanoprecipitation technique with P188 as stabilizer	Size (PCS), zeta potential measurement (electrophoretic mobility) and PDI	63.3 ± 2.82 nm/ PDI = 3.35 ± 0.1
**Liposomes**							
[1]	BNZ-loaded polymerosomes	PEG thioacetate, PEG mesylate, propylene sulfide, BZN and tetrahydrofuran	BZN	1.5 mg of BNZ with 30 mg of the copolymer (PEG_17_-PPS_60_-PEG_17_)	Anionic polymerization, methanol precipitation, and thin film rehydration.	NMR spectroscopy and gel permeation chromatography, TEM, DLS, LC-MS, liquid chromatography–coupled mass spectrometry, SEM. Loading efficiency, EE, size, zeta potential, and morphology	115 nm/ PDI = 0.11 ± 0.02.
**Others Nanocarriers**							
[22]	CaCO_3_ nanoparticles containing BZN (BZN@CaCO_3_)	Calcium chloride, Pluronic F-68, Sodium carbonate and BNZ.	BZN	1 mg of BZN in a solution A containing 50 mL of calcium chloride which has been mixed with a solution B containing 50 mL of sodium carbonate and 50 mL of sodium citrate	Emulsification	Zeta potential, AFM, IR, and UV-Vis spectrophotometry.	27.83–64.01 nm
[23]	MOF nanoparticles coupled to EP (MOFs-EP)	Zn (NO_3_)_2_·6H_2_O, L1, L2 and	EP	50 mg of EP with 100 mg of Zn-MOFs	Heating and cooling for crystal formation (synthesis of MOF nanoparticles). Sensitized photo-oxygenation in methanol with eosin (synthesis of EP). Mechanochemistry for coupling.	IR, XRD, TEM, and SEM	28.67–80.44 nm

Key: AFM = Atomic Force Microscopy; BZN = Benznidazole; C_4_H_4_O_3_ = Succinic anhydride; CHN = Elemental Analysis; CS = Chitosan; CTAB = Hexadecyl trimethylammonium bromide; DETC = Sodium diethyldithiocarbamate, DFT = Density Functional Theory Techniques; DL = Content; DLS = Dynamic Light Scattering (or Dispersion); DSC = Differential Scanning Calorimetry; D-Stroke = Dispersion Index; DTNB = 5,5′-dithiobis(2-nitrobenzoic acid); EDS = Energy Dispersive X-ray Spectroscopy; EDTA = Ethylenediamine Tetra-acetic Acid; EE = Encapsulation Efficiency; EFTEM = Energy-Filtered Transmission Electron Microscopy; EP = Ergosterol peroxide; EtOH = Pure ethyl alcohol; FTIR = Fourier Transform Infrared Spectroscopy; FTIR-ATR = Infrared Absorption Spectroscopy; GPTMS = 3-glycidyloxypropyl trimethoxysilane; HCl = Hydrochloric acid; HPLC = High Performance Liquid Chromatography; HPLC-UV = High-efficiency Liquid Chromatography with Ultraviolet Detection; ICP-OES = Inductively Coupled Plasma—Optical Emission Spectroscopy; ID = Size distribution; IR = Infrared Spectrometry; LYC = Lychnopholide; MSA = Mercaptosuccinic Acid; MOF = Metal-Organic Frameworks; NaNO_2_ = Sodium nitrate; NaOH = Sodium hydroxide; NLC = Nanostructured Lipid Carriers; PBS = Phosphate Buffered Saline; PCL = Poly-ε-caprolactone; PCS = Photon Correlation Spectrometry; PDI = Polydispersity Index; PEG = Polyethylene glycol; PLA = Polylactic Acid; PLGA = Poly (Lactic Co-Glycolic Acid); PLA-PEG = Polylactic Acid/Polyethylene glycol; SEM = Scan Electron Microscopy; SLN = Solid Lipid Nanoparticles; SS NMR = Solid-State Nuclear Magnetic Resonance; TEM = Transmission Electron Microscopy; TEOS = Tetraethyl orthosilicate; TPP = Sodium Triphosphate; UA = Ursolic acid; UPLC = Ultra-Performance Liquid Chromatography; XPS = X-ray Photoelectron Spectroscopy; XRD = X-ray Diffraction; Zn(NO_3_)_2_·6H_2_O = Zinc nitrate hexahydrate.

**Table 4:** In vitro assays.

Reference	Cells	Anti-Epimastigotes	Anti-Trypomastigotes	Anti-Amastigotes	CC_50_—Cells	IC_50_—Epimastigotes	IC_50_—Trypomastigotes	IC_50_—Amastigotes
**Polymeric Nanoparticle**								
[4]	LLCMK2 cells (kidney epithelial cells from Macaca mulatta, CCL-7). Cytotoxicity (MTT) in peritoneal macrophages.	Antiproliferative effect against epimastigotes by direct count in a hemocytometer. Scanning and transmission electron microscopy of epimastigotes.	Effect on trypomastigotes viability in hemocytometer under a light microscope.	-	400 ± 5.7 μg/mL	75.0 ± 6.5 μg/mL	IC_50_ = 25.0 ± 5.0 μg/mL	-
[7]	Potential toxicity of LIC by calcium homeostasis in isolated cardiomyocytes from healthy mice	-	-	-	-	-	-	-
[24]	MTT against three cell lines: RAW (ATCC number TIB-71), derived from macrophages, 3T3 (ATCC CRL-1658), derived from fibroblasts, and Vero (ATCC CCL-81), derived from renal epithelial cells.	Induction of ROS production by parasites in the epimastigote form exposed to DETC nanoparticles.	Antiparasitic activity against different of *T. cruzi* strains in trypomastigotes determined by resazurin reduction and ROS production.	-	-	-	(Dm28c strain) Nanoparticle IC_50_ = 15.47 ± 2.71 μM. Free BZN = 70.58 ± 6.87 μM. (Y strain) Nanoparticle IC_50_ = 45.15 ± 5.44 µM. Free BZN = 85.24 ± 5.22 μM. (Bolivia Strain) Nanoparticle IC_50_ = 47.89 ± 3.98 μM. Free BZN was 79.78 ± 6.18 μM.	-
[29]	Cytotoxicity by the resazurin method in LLCMK2 fibroblasts and *Trypanosoma cruzi* cells.	-	Cytotoxicity in *Trypanosoma cruzi* cells in the trypomastigote form by the resazurin method.	-	-	-	-	-
**Lipid Nanoparticles**								
[2]	WST (water soluble tetrazolium test) cytotoxicity in L-929 murine fibroblasts (NCTC 929 clone, ECACC 88102702) and human hepatocellular Hep G2 cell line (American Type Culture Collection (ATCC))	Biological activity of SLNs, NLCs, and liposomes against epimastigotes. Growth inhibition assays.	Growth inhibition assays.	Growth inhibition assays.	-	SLNs (5, 10, 20): 48.8 ± 14.3 μM–123.9 ± 19.7 μM. NLCs: 41.3 ± 9.9 μM–256.0 ± 19.9 μM. BNZ: 17.7 ± 2.1 μM. Liposomes: It was not viable.	NLCs 20″: 17.6 ± 3.3 μM. BNZ: 0.8 ± 0.4 μM	NLCs 20″: 17.6 ± 3.3 μM BNZ: 0.8 ± 0.4 μM
[10]	Cytotoxicity by flow cytometry in spleen cells isolated from C57BL/6 mice previously cultured in fibroblasts (LLCMK2)	-	Evaluation of the trypanocidal activity of free H2bdtc, H2bdtc-SLNs, and BZN after 24h of incubation with trypomastigotes forms	-	-	-	Free H2bdtc—IC_50_ of 0.50 ± 0.12 μM, H2bdtc-SLNs of 1.8 ± 0.18 μM and BZN of 0.50 ± 0.39 μM.	-
**Mesoporous Silica Nanoparticles**								
[3]		Biological assays—trypanocides for the epimastigotes forms of the *T. cruzi* CL Brener strain.	-	-	-	-	-	-
**Silver Nanoparticles**								
[5]	Cytotoxicity (MTT) on murine macrophages (RAW 264.7 ATCC TIB-71) and mouse fibroblasts (3T3 ATCC CCL-92).	Evaluation of the antiparasitic activity of nano xylan by colorimetric MTT. Flow cytometry.	-	-	-	-	-	-
**Polymeric Micelles**								
[27]	Quantification of reactive oxygen species (ROS) and *T. cruzi*-specific antibody production in cardiac tissue inflammation in Vero cells (African green monkey renal epithelial cells) by fluorescence assay.	-	-	-	-	-	-	-
[28]	Toxicity (MTT) in Vero cells.	-	-	Amastigote growth inhibition assay in mouse cardiac myocytes (CMs) and Vero cells.	-	-	-	-
**Liposomes**								
[1]	It did not assess cell cytotoxicity. Mouse myoblast H9C2 cells for trypanocidal effect	-	Trypanocidal efficacy against trypomastigotes	Trypanocidal efficacy against amastigotes	-	-	BNZ = 55.87 ± 11.39 μM. BNZ-PSs = 56.06 ± 12.21 μM	IC_50_ de BNZ = 33.07 ± 8.17 μM BNZ-PSs = 3.51 ± 0.79 μM.
**Other Nanocarriers**								
[22]	Cytotoxicity (MTT) in LLCMK2 mammalian cells (Rhesus monkey kidney epithelial cells)	Trypanocidal effect against epimastigotes (6.25–50 μg/mL)	Trypanocidal effect against trypomastigotes	Trypanocidal effect against amastigotes—Bz-NP (8.7 and 17.4 μg/mL) and Bz (56.7 and 113.4 μg/mL)	55.35 ± 9.03 µg/mL for encapsulated BZN and 160.4 ± 75.09 µg/mL for free BZN	Encapsulated BZN—24 h of 8.72 μg/mL, 48 h of 8.02 μg/mL and 72 h of 4.8 μg/mL. For free BZN: 24 h of 56.7 μg/mL, 48 h of 15.91 μg/mL and 72 h of 4.3 μg/mL	Free BZN LC_50_: 66.9 ± 20.3 µg/mL. Encapsulated BZN: LC_50_ 1.77 ± 0.58 µg/mL	BZN encapsulated IC50 (8.72 µg/mL). Free BZN IC_50_ (56.7 µg/mL)
[23]	Cytotoxicity (MTT) on NIH-3T3 mammalian cells (isolated mouse fibroblast cell line), J774A.1 (monocyte, mouse macrophage), and Vero (African green monkey renal epithelial cells).	Trypanocidal activity at the following concentrations: 5, 10, 20, 50, 100, and 500 µg/mL of MOFs and MOFs-EP. In addition to MOF suspensions in culture media.	Trypanocidal activity at the following concentrations: 5, 10, 20, 50, 100, and 500 µg/mL of MOFs and MOFs-EP. In addition to MOF suspensions in culture media.	-	(MOFs) CC_50_ of 392.0 µg/mL for NIH3T3 cells. CC_50_ of 593.6 µg/mL for J774A.1 cells. CC_50_ of 1030.0 µg/mL for Vero cells. (MOFs-EP)	Results not shown because they are like those found for trypomastigotes	(MOFs-EP) IC_50_ of 4.81 μg/mL and 3.0 μg/mL for 24 and 48 h	Not performed.

Key: CC_50_ = Cytotoxic drug concentration required to kill 50% of the cells; DETC = Sodium diethyldithiocarbamate; H2bdtc = 5-hydroxy-3-methyl-5-phenyl-pyrazoline-1-(S-benzyl dithiocarbonate); IC_50_ = Inhibitory concentration of the drug capable of killing 50% of the parasites; SI = Selectivity index; LIC = Lychnopholide; MOFs = Metal–Organic Frameworks; NLCs = Nanostructured Lipid Carriers; ROS = Reactive Oxygen Species; SLNs = Solid Lipid Nanoparticles, WST = Water Soluble Tetrazolium Test.

**Table 5:** In vivo assays.

Reference	Animal Model	Infection	Treatment	Control Group	Assays
**Polymeric Nanoparticles**					
[7]	Male C57BL/6 mice aged seven weeks old		Daily intravenous injections of free LIC solution (2.0 mg/kg/day; 8 mice), LYC loaded in biodegradable polymeric NP (LYC-NP; 2.0 mg/kg/day, 10 mice), blank NP (10 mice), and vehicle (control group; 10 rats) for 20 consecutive days	Control group (vehicle, 10 mice)	Transthoracic echocardiography. Single-cell and real-time Ca^2+^ contracting—imaging. Studies of the cardio-toxicological effects of LYC on cardiac function. Effect of LYC encapsulation on NP.
[8]	Swiss mice aged between 28 and 30 days old and weighing from 20 to 25 g	Infection by trypomastigotes injection 104—Intraperitoneal route	LYC—2 mg/kg/day for 10 and 20 days in the acute phase of the disease. Multiple doses of BZN, free LYC, LIY-PCL NP, unloaded NP, LYC-PLA-PEG NP, and DMA-PEG 300 (i.v. control solution) according to the strain and days. Route—intravenous	Untreated control (infected but untreated), NC unloaded control (UN-NP), and DMA-PEG 300 control (control solution)	Parasitemia level by the Filardi and Brener method. Evaluation of parasitological cure by parasitological methods (examination of fresh blood, blood culture, and PCR in peripheral blood) and conventional serology.
[25]	C57BL/6 mice (Female and male mice aged eight weeks old)	10,000 Trypomastigotes/mouse—Intraperitoneal route	BZN + PLG-Cur. 0.15 mL by gavage/day. BZN. One quarter of 25 mg/kg/day. Cur nanoparticle—One 200 mg/kg daily dose	Uninfected and infected but untreated	(Quantitative PCR). Atrial natriuretic peptide measurements (ELISA). Serum creatine kinase (CK) activity (NADP-reduction photometric assay). Histopathological analysis of the heart (hematoxylin, eosin, and Masson’s trichrome). Myocardial cytokine and chemokine concentrations (ELISA). Enzyme activity by gelatin zymography and transmission densitometry.
[29]	Male C57BL/6 mice (20–22 g)	1 × 10^3^ trypomastigotes (Y strain)—Intraperitoneal route	BZN 2.5 μg/animal/day; blank polymeric nanoparticles; Polymeric PN-UA-2 (13.15 μg/animal/day)—Intra retro-orbital route of samples diluted in 50 μL of PBS	Control group not treated (negative control)	Trypanocidal activity, parasitemia by counting trypomastigote forms of the parasite per 5 µL. Liver markers.
[30]	Female Swiss mice aged 28–30 days and weighing 20–25 g.	10,000 trypomastigotes (Y strain) for the acute phase—Intraperitoneal route. 500 trypomastigotes (Y strain) for chronic phase—Intraperitoneal route	For acute phase: LYC (free LYC, LYC-PCL-NP, and LYC-PLA-PEG-NP) 5 mg/kg for 20 days. A group with BZN for a murine model (100 mg/kg) orally via gavage (0.2 mL). For chronic phase: LIC (free LYC, LYC-PCL-NP, and LYC-PLA-PEG-NP) with LYC doses of 5 mg/kg/day. LYC at 2 mg/kg/day. Two groups received daily BZN doses of 100 mg/kg/day (0.1 mL) and 50 mg/kg/day (0.2 mL)—Orally (gavage) or intravenously (rear vein)	Untreated animals (infected and untreated), animals treated with solution excipients (DMA-PEG), and animals treated with blank NP	Therapeutic efficacy by fresh blood test, blood culture, PCR, and enzyme-linked immunosorbent assay (ELISA). Parasitemia level by FBE method, survival rates, blood culture (BC).
[31]	Female Swiss mice (age: 28–30 days; body weight: 20–25 g)	*T. cruzi* VL-10 strain. Acute phase model: 10,000 trypomastigotes—Intraperitoneal route. Chronic phase model: 500 blood trypomastigotes—Intraperitoneal route.	Free LYC (12 mg/kg of body weight/day), LYC-PLA-PEG-NP (8 or 12 mg/kg/day) or BZN at 100 mg/kg/day—via oral route	Infected and untreated	Treatment efficacy was evaluated by fresh blood test, blood culture, PCR, and enzyme-linked immunosorbent assay (ELISA). *T. cruzi* VL-10 strain DTU II. Survival rates and heart histopathology.
**Lipid Nanoparticles**					
[10]	Swiss mice (6–8 weeks old), weighing 20–25 g.	*T. cruzi* Y strain (Type II linage). 2.0 × 10^3^ trypomastigotes—Intraperitoneal route	BZN, free H2bdtc and H2bdtc-SLNs administered orally at 4 μmol/kg (BZN 1.0 mg/kg/day; free H2bdtc and H2bdtc-SLNs 1.4 mg kg/day) per day for 10 consecutive days	Group 1 = PBS infected and untreated	Cytotoxicity and trypanocidal activity of free H2bdtc and H2bdtc-SLNs in Swiss mice (6–8 weeks old). Evaluation of parasitemia and mortality. Measurement of creatine kinase-MB (CK-MB) and glutamic–pyruvate transaminase levels. Histological analysis to assess inflammatory infiltration through light microscopy DP71.
**Polymeric Micelles**					
[26]	Female C57BL/6J mice aged 1 month old.	Chronic model of the *Trypanosoma cruzi* Nicaragua infection. Route—Intraperitoneal with 3000 trypomastigotes.	30 BZN-MP daily doses at 50 mg/kg/day; 30 BZN-MP daily doses at 25 mg/kg/day; 13 BZN-MP intermittent doses at 75 mg/kg; 13 BZN-MP intermittent doses at 50 mg/kg. Intermittent—One dose every 7 days BZN-MP doses Via—Oral gavage	Infected and untreated (only oil)	Induction of immunosuppression and evaluation of parasitemia by DNA amplification. ECGs performed with electrocardiogram, measurement of IgG antibody response by ELISA, histopathological studies.
[27]	Female C3H/HeN mice aged 1 month old.	*Trypanosoma cruzi* Nicaragua. 1000 trypomastigotes. Route—Intraperitoneal	BZN 50 mg/kg for 30 days; BZN-MP 50 mg/kg/day for 30 days; BZN-MP 25 mg/kg/day for 30 days; BZN-MP 10 mg/kg/day for 30 days. Route—oral gavage	Infected without treatment	Monitoring of parasitemia and of the survival rates. Induction of immunosuppression with cyclophosphamide and estimated number of parasites as described by PCR. Analysis of IgG antibody levels by enzyme-linked immunosorbent assay (ELISA). Histopathological studies by microscopy.
[28]	Female C3H/HeN mice aged 1 month old.	1000 trypomastigotes—Intraperitoneal route.	R-BZN and BZN-MPs were dispersed in olive oil and administered to mice via oral gavage. R-BZN 50 mg/kg/day for 15 days; BZN-MPs 50 mg/kg/day for 15 days; BZN-MPs 50 mg/kg/day for 30 days; BZN-MPs 25 mg/kg/day for 15 days; BZN-MPs 25 mg/kg/day for 30 days; BZN-MPs 10 mg/kg/day for 15 days; BZN-MPs 10 mg/kg/day for 30 days; BZN-MPs 50 mg/kg/day for 30 days (uninfected)	Infected without treatment	Assay in the acute phase of infected mice, parasitemia, antiparasitic effect of BNZ-MPs, survival curve during the acute phase, Kaplan–Meier test to differentiate the curves.
**Liposomes**					
[1]	Female BALB/c mice (4–6 weeks old)	Infected with the *T. cruzi* Y strain. 2 × 10^3^—Intraperitoneal route.	PS 0.3 mg/mL by i.v. injection; BZN 100 mg/kg/day orally for 14 days; BZN-PS i.v. at a BZN dose of 1.5 mg/kg (2 doses); BZN-PS i.v. at a BZN dose of 0.15 mg/kg (2 doses), BZN-PS at a BZN dose of 0.03 mg/kg (2 doses)	Infected and untreated	Monitoring of parasitemia, cardiac parasitosis quantified by quantitative PCR, cardiac inflammation, and cardiac histology. Hepatotoxicity. Assessment of toxicity and determination of serum alanine aminotransferase.

Key: BZN = Benznidazole; Cur = Curcumin; ECG = Electrocardiogram; ELISA = Enzyme-Linked Immunosorbent Assay; H2bdtc = 5-hydroxy-3-methyl-5-phenyl-pyrazoline-1-(S-benzyl dithiocarbonate); IgG = Immunoglobulin G; LYC = Lychnopholide; NC = Nanocapsule; PBS = Phosphate Buffer Sodium; PCR = Polymerase Chain Reaction; SLNs = Solid Lipid Nanoparticles.

## Data Availability

Data sharing not applicable.
